# Regional food self-sufficiency potential in the European Alpine space

**DOI:** 10.1038/s41598-024-60010-z

**Published:** 2024-04-25

**Authors:** Caroline Pecher, Thomas Marsoner, Erich Tasser

**Affiliations:** Institute for Alpine Environment, Eurac Research, Drususallee/Viale Druso 1, 39100 Bozen/Bolzano, Italy

**Keywords:** Sustainability, Agroecology

## Abstract

The sustainability of the food system needs to be improved, including shortening supply chains and promoting the consumption of regional food. Here, we explore the current potential for regional food self-sufficiency in the European Alpine space by calculating the current regional food/feed energy balance, deriving the regional per capita land footprint based on current food/feed consumption rates, and modelling the current potential for regional food/feed self-sufficiency. We show that 59% of the 560 Pcal of energy currently available in the study area comes from domestic production, and almost 60% of the energy is used for livestock consumption, with high regional variability. The resulting land footprints range from 2301 to 2975 m^2^ cap^−1^ y^−1^. Taking into account changes in cropping patterns, partial intensification, but no expansion of agricultural land, the European Alpine space could produce 89% of its current food demand domestically, with high regional variability due to population density, availability of agricultural land, crop yields, climatic conditions and dietary habits. These findings highlight the potential and limitations of regional mountain food systems and call for new strategies to improve sustainability. Reducing the current high consumption of animal products would reduce the land footprint and increase the potential for food self-sufficiency.

## Introduction

The current food system faces the challenge of supplying a continuously growing world population with food ^1^. However, the current food system is not sustainable. The food system alone is responsible for one-third of global GHG emissions ^2^, the depletion of water resources ^3^, land-use changes ^4^, and the loss of biodiversity ^5^. The livestock sector plays a major role in this scenario, using approximately 75% of the earth’s agricultural area for feed production ^6^. Moreover, this vulnerable system, in which trade dependency has been strongly increased during the last decades ^7^, is affected by a continuous decline in agricultural land ^8^, negative effects of climate change ^9^, or crises such as the COVID-19 pandemic ^10^ and the war in Ukraine ^11^.

Several actions are underway to make the global food system more sustainable. The UN Sustainable Development Goals (SDGs) 2 and 12 set the most important targets for improving the current food system and ensuring sustainable exploitation of related resources ^12^. In the European Union (EU), several initiatives and strategies, such as the Green Deal ^13^ and the Farm to Fork strategy ^14^, highlight the urgent need to increase sustainability, inter alia, by shortening supply chains and promoting the consumption of regional food. However, despite the EU being “largely self-sufficient for food” ^15^, it is to a large share co-responsible for deforestation in other parts of the world due to current types of agriculture ^16,17^. Moreover, the EU’s food system is vulnerable to crop failures or trade disruptions due to natural hazards, health emergencies, or sociopolitical conflicts. Recently, the war in Ukraine led to immediate measures to alleviate the pressure on the EU food system, including the temporary recultivation of abandoned land ^15^.

A number of studies have investigated the food self-sufficiency potential of countries and regions ^18–25^. Beltran-Peña et al. ^18^ assessed global food security through the twenty-first century under various scenarios, revealing a decline in self-sufficiency despite sustainable agricultural intensification, while suggesting sufficient global food production under a sustainability scenario. Fader et al. ^19^ examined globally the spatial decoupling of agricultural production and consumption finding that 16% of the world population are currently heavily reliant on international food trade with a potential increase to 51% by 2050. Pradhan et al. ^21^ identified the potential for local food supply by calculating the food-self-sufficiency globally at 5’ resolution finding that only about 2 billion people are locally self-sufficient, a number that could be increased by 1 billion people. Menconi et al. ^23^ introduced a new concept of the Food Self-Sufficiency Index (FSSI), which assesses self-sufficiency based on locally chosen diets and re-localized food production using crop suitability maps, which had already been applied at national and regional levels in Menconi et al. ^24^ and Stella et al. ^25^. Kaufmann et al. ^22^ conducted six different self-sufficiency measures for the year 2012 for 226 NUTS 2 regions in the EU, two for livestock products, two for crops, one for grassland, and one overall measure including crop/grassland products, by modelling regional food and feed production and consumption without trade. According to their results, self-sufficiency levels in EU regions differ depending on the measure under study, whether only livestock products, crops or grassland, or a combination of all, are considered. They found, among other things, that large parts of Central and Western Europe, with the exception of the densely populated regions of western Germany and the Benelux countries and the central and southern Alpine region, were self-sufficient in food and feed from crops and grasslands in 2012 ^22^.

The European Alpine Space is a transnational high mountain region spanning six countries in Central and Western Europe, which has experienced significant changes in land use and agricultural practices in recent decades due to globalization, ranging from intensification of favourable sites to abandonment of less favourable sites ^26^. It is characterized by cultural diversity and it encompasses different climatic zones, land-cover types, and site conditions. The region is a hotspot for the provision of ecosystem services such as fresh water for drinking, carbon sequestration, biodiversity conservation and outdoor recreation for its foothills and adjacent lowlands, which are home to some of Europe’s largest cities ^27^. According to Kaufmann et al. ^22^, a large part of the European Alpine region, especially in the north, south and east, is self-sufficient in terms of milk and meat from ruminants and in food and feed from crop- and grassland. In terms of potential food and feed self-sufficiency, only part of the northern and north-eastern Alpine region is considered to be self-sufficient in 2012 ^22^. Although numerous studies have developed scenarios for global food self-sufficiency, including the potential decoupling of production from trade, many of them do not provide detailed results at the regional level. This is especially true for heterogeneous and diverse regions such as the European Alpine Space. The study by Kaufmann et al. ^22^ with its various self-sufficiency measures for EU NUTS 2 regions provides valuable information at the regional level, but does not consider potential changes to the current food system to improve its self-sufficiency performance, e.g. through demand-driven production. Moreover, the use of only one reference year and the lack of Switzerland pose a challenge for research issues specific to the Alpine Space region.

This study aims to model potential food self-sufficiency in the European Alpine Space at the regional level by taking a holistic approach. We take into account a large selection of crop classes, local site conditions, climatic conditions, the potential for land-use changes, the use of crop sequencing, food consumption habits per country, using data from a range of reference years. In a first step, we quantify the current energy balance of the food system in the European Alpine Space at the regional level consisting of production, import/export, and consumption. In a second step, we quantify the per capita land footprint of the currently consumed food and feed by tracing back current food consumption step by step to its land footprint. In a final step, we model the demand-driven potential for regional food self-sufficiency at the NUTS2 level, i.e., the capacity of the European Alpine Space to feed its population considering only the existing site conditions and eating habits. To determine only the production capacity of the study region, we use a strictly demand-driven modelling approach decoupled from economic incentives and global trade.

## Results

### Current regional food and feed balance

**Figure 1:**
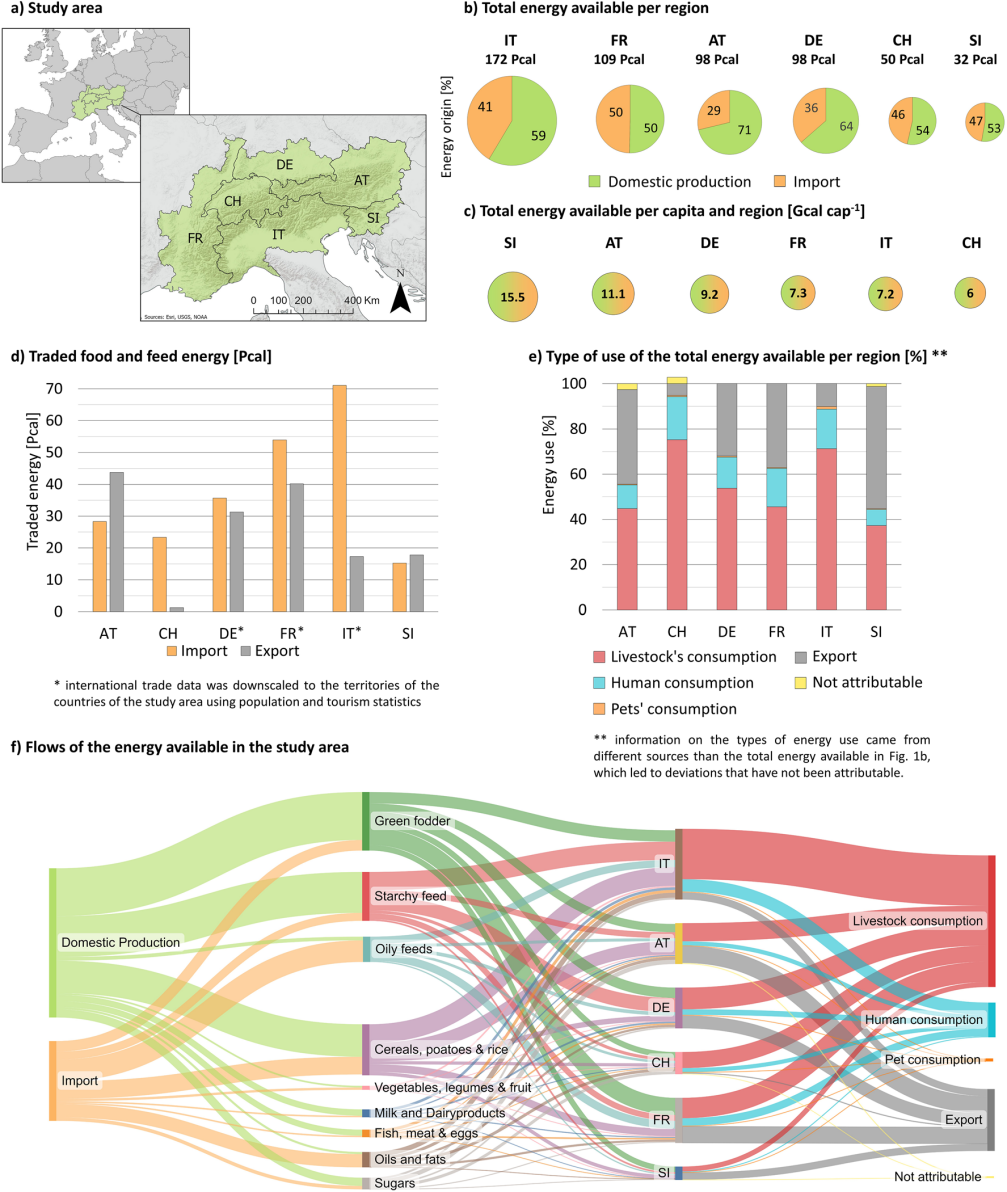
Study area and its total available nutritional energy per year between 2014 and 2018: (**a**) the study area includes the whole of Austria (AT), Slovenia (SI) and Switzerland (CH), as well as parts of France (FR), Germany (DE) and Italy (IT); in this study, the country codes refer to the territories of the countries within the study area; (**b**) the total nutritional energy available per region (import was downscaled from national data for DE, FR, IT); (**c**) the average nutritional energy available per capita; (**d**) comparison of imports and exports of food and feed energy (both downscaled from national data for DE, FR, IT); (**e**) current use of the available nutritional energy; (**f**) flows of the available energy. Import is defined as imports from other regions within the same country as well as from other countries. Exports are defined as exports to other countries. Since production, trade and consumption data were obtained from different sources, a completely balanced food and feed energy balance was not always possible (cf. Fig. 1e).

In our study area (cf. Fig. 1a), an average nutritional-energy amount of ca. 560 Pcal was available annually between 2014 and 2018 (cf. Fig. 1b). 59% of this energy came from domestic production, and the remainder was imported (Fig. 1b, f). More than 57% of the energy was consumed by livestock, ca. one-quarter was exported, and only 15% was consumed by humans (Fig. 1e, f). Pet consumption was negligible, accounting for less than ca. 1% of the total energy available. AT and DE domestically produced more than 60% of the total energy available in their regions, and FR had the lowest share of domestic production at 50%. AT and SI were net exporters, whereas IT, CH, and FR were net importers (cf. Fig. 1d). Green fodder, starchy feed, most animal-based products, sugars, cereals and potatoes were mainly produced in the study area (cf. Fig. 1f). In contrast, most oily and high-protein feedstuffs and oils and fats for human consumption had to be imported. Given the different sizes of the regions, IT had the highest share of energy available per country (31%), and SI had the lowest share (6%, Fig. 1b). However, the energy available per capita was highest in SI and lowest in CH, FR, and IT (Fig. 1c). The per capita human consumption of food including waste was very similar across the regions of the study area, at ca. 1.2 Gcal cap^−1^.

### Current regional consumption land footprint

**Figure 2:**
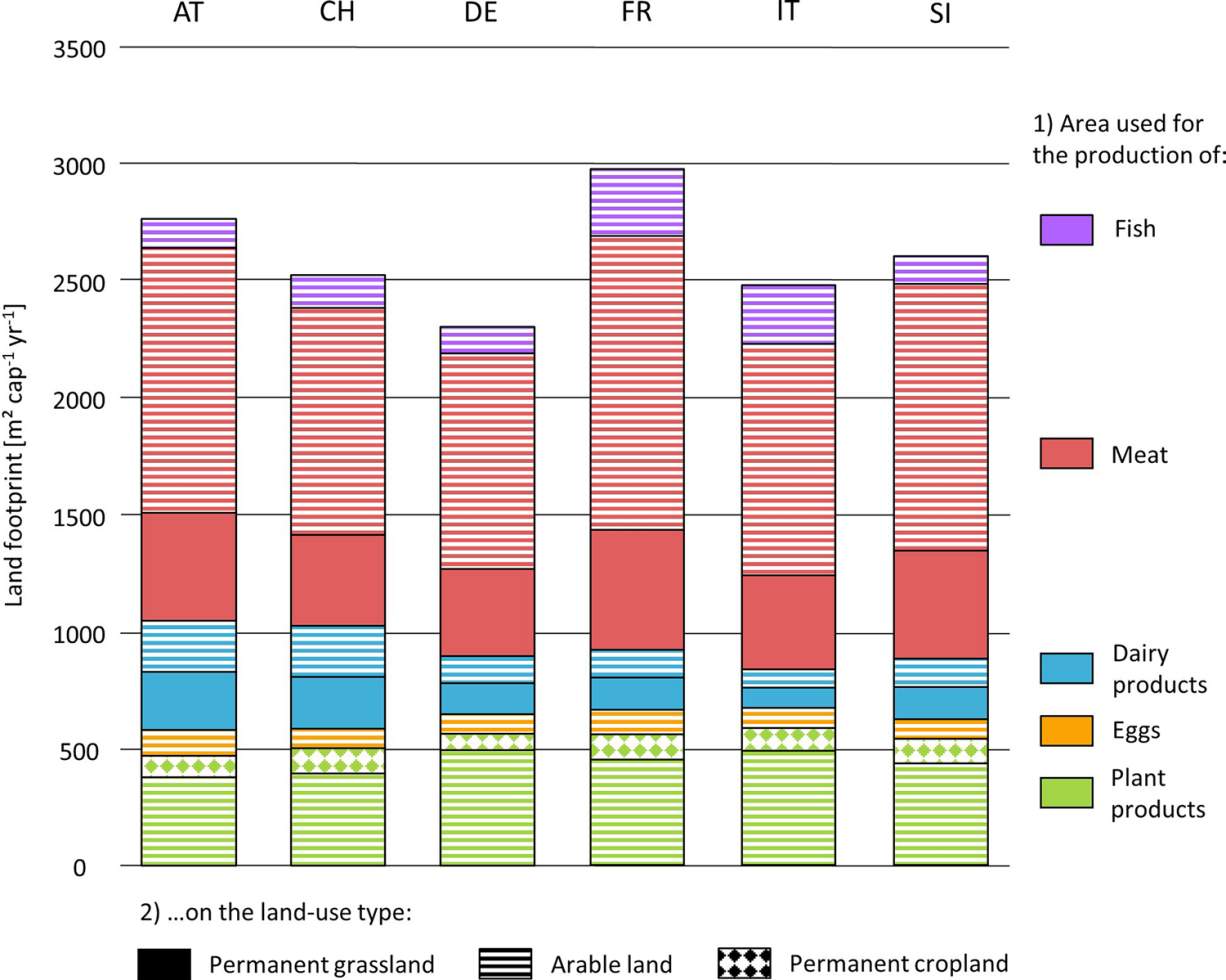
Current land footprint [m^2^ cap^−1^  yr^−1^] based on the average regional per capita food consumption of residents and tourists for 2014–18 ^28,29^ weighted by the average regional food/feed production potential derived from yield data and, for animal products, herd composition, energy demands and feed composition by herd class (cf. Methods chapter 4.2.2). The colours represent the areas [m^2^] that were needed for the production of the single food-product groups, whereas the hatching indicates the land-use types needed for the production.

Between 2014 and 2018, the net food consumed by residents and tourists resulted in an annual per capita land footprint between 2301 m^2^ (DE) and 2975 m^2^ (FR, cf. Fig. 2), excluding regional food waste (cf. 4.2.2; Appendix 20). Most of the land needed was arable land (73%), followed by permanent grassland (23%) and cropland with permanent crops (4%). More than half of the land was used for the production of meat (57%), the rest was used for the production of plant products (21%), dairy products (12%), fish (7%) and eggs (3%). The differences in the land footprints between regions were caused by different food-consumption habits and, more importantly, by differences in the production capacity per used agricultural area (UAA; cf. Fig. 2, Appendix 3). The largest difference was found in the land footprints for meat production, which was 411 m^2^ per capita larger in FR than in DE, mainly due to a lower production capacity.

### Regional potential for food self-sufficiency

**Figure 3:**
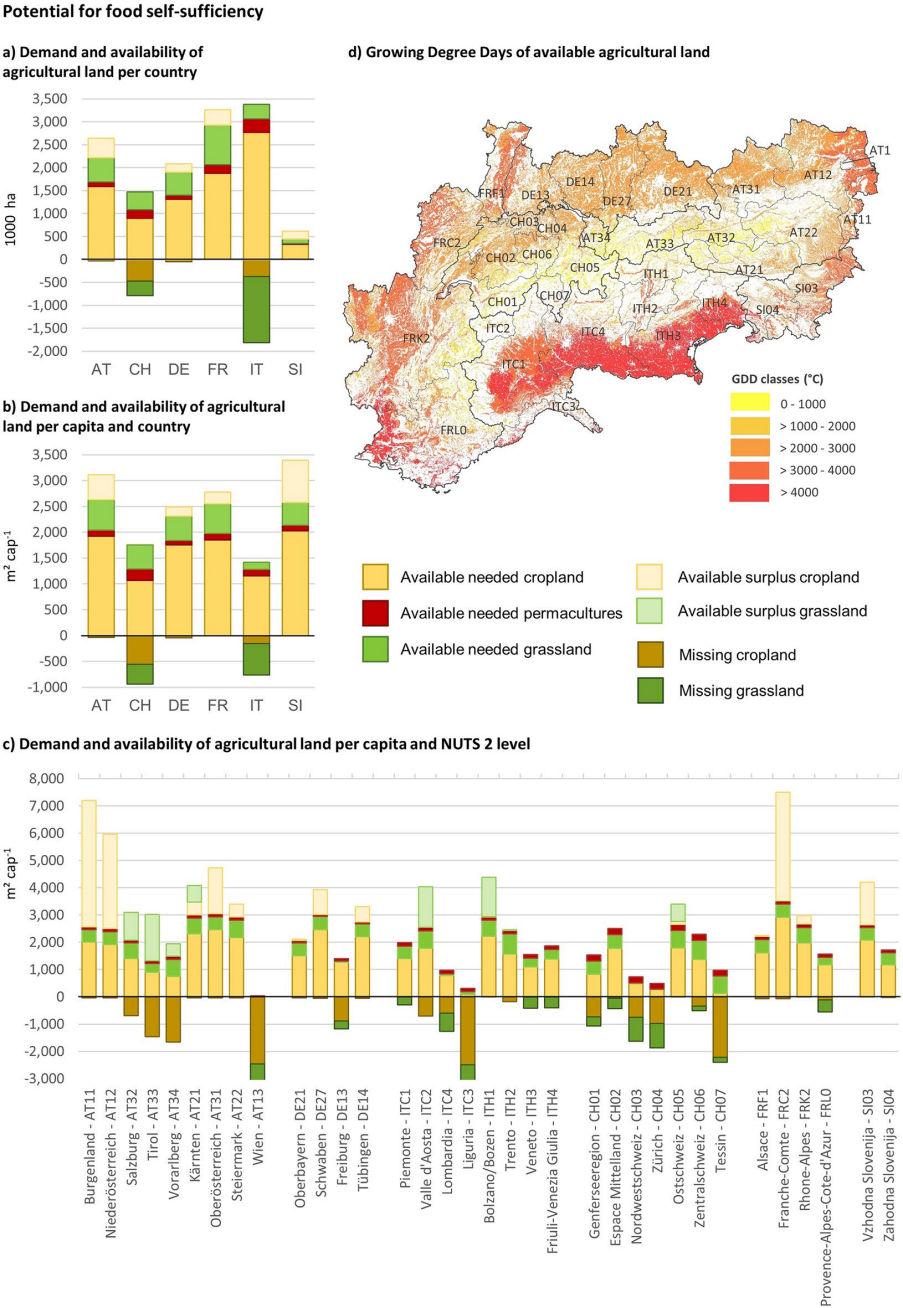
Potential for food self-sufficiency based on the modelled growing degree-days (GDDs, cf. chapter 4.2.3) for the current food consumption per national territory and NUTS 2 region of the study area.

Our analyses revealed that the study area (cf. Fig. 1a and chapter 4.1) has the potential to cover 89.1% of its current food demand by domestic production (cf. Fig. 3). AT, FR and SI have the highest potential to reach food self-sufficiency. Although AT has more agricultural land than it needs to reach 99% of its food self-sufficiency potential, some crops such as rice and citrus fruits cannot be grown due to insufficient GDDs. At the same time, AT, DE, FR, and SI have a surplus of cropland, which could be used for the production of export goods or as biodiversity compensation areas. At 65%, both CH and IT have a comparatively low self-sufficiency potential. While cropland would have a higher priority in such an optimized regional production scenario, grassland would be missing in CH by 45% and in IT by 82%.

At an even more local level, the differences in available land use types, climatic conditions and population densities are clearly visible. The differences between the NUTS 2 regions are considerable, ranging from an available cropland surplus of approximately 4500 m^2^ per capita in Burgenland (AT11) to a deficit of approximately 2400 m^2^ per capita in Vienna (Wien AT13) or Liguria (IT42). Overall, a total of 10.9% of the total food demand would have to be imported.

## Discussion and conclusion

In this study, we quantified the current energy budget of the food system in a European mountainous cross-border region, identified the per capita land footprint of the currently consumed food accounting for livestock feed, and modelled the potential for regional food self-sufficiency assuming demand-driven production. Our study offers an advanced approach for quantifying the regional potential for food self-sufficiency, and provides another level of detail to the ongoing debate on food self-sufficiency ^18–23^, as it is based on a holistic modelling approach that takes into account the potential for land use changes, including crop-sequences, local site and climatic conditions, and data from a five-year range of reference years. Another unique feature of our study is that it assesses the potential for food self-sufficiency in a diverse mountainous region with strong regional differences in climatic and topographic conditions and, consequently, in agricultural production and its outputs. Combined with strong regional differences in population size, as well as national differences in food consumption, our study is able to identify different levels of food self-sufficiency potential and a variety of drivers behind them. According to our results, the European Alpine space has the potential to produce 89.1% of its current food demand domestically. 44% of all NUTS 2 regions under study would be self-sufficient at more than 95%. The average self-sufficiency potential of the NUTS 2 regions is 75%.

Informed policy decisions require a solid understanding of the potential for increasing regional food self-sufficiency. A crucial initial step in this process, which our results can be seen as, is the assessment of regional food self-sufficiency, which reveals not only the potential but also the limits of regional food systems, which are often unclear in today’s context. The interconnectedness of food systems with international trade can obscure unsustainable practices, allowing them to persist unnoticed for longer periods of time, and raises concerns about food security in the event of trade disruptions ^30^. Establishing a demand-driven food production system at the regional level can increase food production diversity, thereby reducing the risk of supply shortages and the overreliance on a limited range of products to maintain self-sufficiency ^31^. Consequently, improving the agrobiodiversity and resilience of the regional food production system becomes possible ^14,23^.

Complete food self-sufficiency may not be a realistic ^32^ and always desirable ^2^ goal. For instance, while transport is known to be a significant contributor to GHG emissions from the food sector ^33^, the primary sources of the food sector’s GHG emissions are agricultural production, land use and land-use change ^2^. This suggests that regions with less favourable location factors may have less sustainable agricultural performance than regions with more favourable location factors, which may make importing certain foods from these regions a more sustainable option than local production. However, regions with suitable natural resources to achieve food self-sufficiency but currently heavily reliant on food imports as a net deficit, would have the opportunity to reduce their environmental impact by increasing domestic production, in line with the objectives of the Farm to Fork Strategy ^14^.

In monetary terms, the whole EU has recorded an increasing trade surplus from exports and imports of agricultural products since 2012, with a surplus of almost €50 billion in 2021, mainly due to a higher monetary value of refined exported products ^34^. Refining raw products is associated with high energy losses, e.g., from animal feed to animal products or from cereals to alcoholic beverages ^35^, which contributes to high amounts of energy transformed within the study area (cf. Fig. 1f). The consequences of this trade-driven system extend far beyond the EU and the study area. Food and feed imports and exports can bring socioeconomic benefits to non-EU countries of origin and destination ^36^. However, food imports from non-EU countries are also known to have negative socioenvironmental impacts, including deforestation, soil degradation, water depletion, loss of biodiversity, or overfishing in the countries of origin ^3,17,37^. The EU aims to identify exit strategies from several unsustainable practices related to the trade of raw products from outside the EU ^16,38^, but they may not be effective because of inadequate controls or minimal sanctions ^37^.

According to our results, regional differences in current land footprints are closely linked to diets and local yields. Large energy losses in the conversion from primary biomass to final animal products ^35^ in association with a high consumption rate of animal products has undoubtedly the greatest impact on the land footprint in our study (cf. Fig. 2). Over the last decades, livestock farming in the Alps has evolved from a resource-driven activity, tied to local conditions and environments (e.g. feed availability, local breeds), to a demand-driven activity decoupled from local conditions with an increase in the number of animals per farm, indoor production systems, greater use of specialized high-yielding cattle breeds and the use of off-farm concentrates as feed ^39^. While this trend has had positive impacts on outputs production and labour efficiency it has also had negative impacts on animal welfare, local and global ecosystems, and biodiversity. Negative consequences include the land abandonment of high alpine grasslands leading to a decline in biodiversity ^40^. The heightened reliance on off-farm concentrates as feed, coupled with increased livestock numbers and production volumes, contributes to increased greenhouse gas emissions and negatively affects the global nitrogen cycle ^17,41,42^.

Our findings suggest that there is untapped potential for many regions to improve their food self-sufficiency through changes in cropping patterns and partial intensification, without expanding the UAA. However, it is crucial to consider the potential environmental impacts associated with such intensification. For example, the conversion of grassland to arable land can lead to a loss of soil organic carbon, contributing to increased GHG emissions and other unintended consequences ^43^. In addition, our results provide some new insights into the many drivers behind the differences in self-sufficiency levels between regions. Population, the availability of agricultural land combined with climatic conditions and food-consumption patterns all influence the regional self-sufficiency potential:

*Population:* Historically, the availability of locally produced food has strictly limited population growth and caused many migration flows, but the Green Revolution, agricultural intensification, and globalization have largely removed this limiting factor ^19,44^. Our results highlight those regions that are exceeding their production limits and are highly dependent on food and feed imports. Even if agricultural productivity were to increase and adapt to regional demand, these regions would not be able to feed their populations today. AT and CH best illustrate the strong influence of population density on a region's potential for food self-sufficiency. Although AT has the highest per capita land demand for animal-based products, as a result of high consumption patterns and average production yields compared to other regions in the study area, it also possesses the highest potential surplus of cropland due to its relatively low population density. CH, in contrast, with more than twice the population density (208 inhabitants km^-2^) of AT (103 inhabitants km^-2^) ^29^, has a high import dependency, which is to some degree also influenced by slightly different topographic conditions.

*Agricultural land and Climate*: The influence of agricultural land combined with climatic conditions on the potential for regional food self sufficiency is complex. However, our results for the Italian Alpine region provide some insights. The agricultural area here has the highest GDDs, allowing one to three crop sequences per year on arable land; this fact, combined with the lowest footprint for livestock products (mainly due to a lower consumption of animal products), allows Italian regions to have the lowest land footprint. However, the higher share of consumption of vegetables with high GDD requirements, such as aubergines or artichokes, somewhat limits this climatic potential for increased productivity (cf. Appendix 2). The influence of local production capacities on food self-sufficiency can be seen in the German Alpine region: due to a comparatively high grassland productivity resulting from favourable climatic and topographic conditions ^45^, consumption of animal products is less land-intensive in this region than in others (cf. Fig. 2).

*Food consumption:* As with the current land footprint, the potential for food self-sufficiency is highly dependent on diets. Currently, high consumption of meat is responsible for 57% of the land footprint and significantly reduces food self-sufficiency ^35^. However, our research also highlights the role of plant-based food, as illustrated by a comparison between the northern and the southern regions in the Alpine space. The choice of specific food crops is particularly important due to differences in yield, GDD requirements, and the potential for crop sequencing. The diet in the northern Alpine regions is characterised by a higher consumption of products with low climatic requirements and high area yields (e.g. potatoes, rapeseed oil). Conversely, in the southern regions, foods with high GDD requirements and lower yields, such as rice, tomatoes, and olive oil, are more commonly consumed. These examples highlight how regional food choices can change the land footprint. Of course, dietary changes towards a healthy, vegetarian or vegan diet would have a much larger impact ^46,47^. Based on our methodological approach, the effects of alternative diets can also be modelled spatially explicitly for the Alpine space in the future.

Where available and comparable, we have compared our results (cf. Fig. 3) with those of other studies. Fader et al. ^19^ found AT to be independent of external land and water resources while CH and SI resulted as 50–70% import dependent. Our results are consistent with these findings for AT and CH, however, our food self-sufficiency potential for Sl is much higher. The results for FR, IT and DE are only partially comparable with those of existing studies because of divergent areas of observation, but similar results have been obtained by Pradhan et al. ^21^, clearly showing the densely populated mountain regions in the Inner-Alpine-Arc as strongly food-trade dependent. The French Alpine Space shows an overall high potential for food self-sufficiency, and similar to that in Rouxelin et al. ^48^, the NUTS2 region Provence-Alpes-Côte d'Azur (FRL0, cf. Fig. 3) lacks the agricultural land to be fully self-sufficient, even when considering agricultural transformation. In their analysis of current regional self-sufficiency, Kaufmann et al. ^22^ classified Veneto, Styria, Carinthia and Slovenia as current partial net importers, which according to our results would have the potential to become net exporters, while some of the current full net importers (e.g., Lombardy and Liguria) would have only a limited potential to improve their self-sufficiency level. These differences can be explained by slightly different modelling approaches. Our approach is based on optimized production, which is tailored to meet local demand. This approach produces goods that align precisely with local consumption patterns, resulting in a reduction of livestock numbers in some regions. Furthermore, the model considers alterations in cropping patterns to optimize the utilization of seasonal length through strategic crop sequencing and takes into account land use changes within the UAA, resulting in an increase in arable land in mountainous regions. The potential for certain regions to become net exporters stems from factors such as low population density relative to agricultural land availability or high GDD potential.

Our study has some limitations, in particular due to the limited availability of appropriate data at regional level, which required us to identify proxies or workarounds:

- We used international trade data which provided information on imports from and exports to other countries. For DE, FR and IT this information had to be downscaled using population and tourism statistics as we did not have information on domestic trade. Therefore, it was not possible to quantify exports to other regions within the same country for DE, FR, and IT, while domestic imports could be estimated using regional production-consumption based accounting (cf. Methods chapter 4.2.1).
- Our study does not account for regional differences in food consumption, as the national human consumption data has been downscaled to the regional level. Although such differences may be small in smaller countries due to national food retail chains and similar traditions in growing and consuming food, larger deviations from actual consumption can be expected in larger countries where only a smaller proportion of the total population lives in our study area, such as DE, FR, and IT ^49,50^. Despite its limitations, this approach ensures that the data is comparable between the regions of our study area and the aggregated results of regional and national data are consistent ^22^.
- We calculated the current regional land footprint on the basis of local yields, even though consumed food products are produced only in part locally and the footprint of imported goods can vary considerably. We used regional yield statistics as a proxy for crop production potential. This approach may have biased our study, as many agricultural products are currently only grown at a small scale and under very favourable conditions. Thus, site-specific and management-related potentials may have been incorrectly estimated ^51^, as various framework conditions, such as different fertilizer-use intensities or climatic conditions, were currently not empirically covered.
- We differentiated between UAA with a slope < 16° suitable for arable crops, vineyards, and orchards and those with a higher slope suitable for grasslands, although crop and wine production is possible on steeper slopes with higher cultivation effort. In particular, the area under cultivation in Switzerland may have been underestimated based on this restriction.
- Using GDDs, we integrated temperature conditions into our modelling approach, but soil suitability and water availability were not directly considered. In particular, soil data were not available at a sufficiently high spatial resolution. We approximated the actual conditions by using regional yield statistics, which are also influenced by water availability. However, given the dependence of agricultural use in the study area on precipitation and—especially in many southern parts of the study area—on irrigation ^52^, a more comprehensive consideration of water availability would be useful for scenario development.

The main advantages of our approach are the selection of crop classes and the level of detail of our analyses. We took into account a large selection of 48 crop classes, local site conditions, national differences in food consumption, feed and its related production chains, the potential for land conversions from grassland to cropland, and the potential for shifts between different crops and livestock breeds. Additionally, we compiled a comprehensive collection of data and information on food and feed production requirements, including food energy densities, GDD requirements of single food/feed products, herd/feed compositions, and feed demands, which were used to model the agricultural areas needed for the production of plant and animal products. These data are available in the Appendix and are ready to be used in other studies.

Our results suggest that there is a high untapped potential for regional demand-driven food production through partial intensification and land use changes within UAA, even without expanding agricultural land. Even in a comparatively small region such as the European Alpine Space, the variation in local food-based land footprints and potentials for food self-sufficiency can be very large. Small differences in current diets coupled with differences in local production potentials and population densities lead to very heterogeneous results and implications. This finding is important in regard to developing new strategies to improve food-system sustainability, and it clearly shows the limits of local food systems. Given the strong impact of the current high consumption rates of animal products on land footprints, reducing the consumption of these products would probably have the largest impact on both land footprints and the potential for food self-sufficiency. It would therefore be very useful to study the impact of dietary changes on the regional potential for food self-sufficiency in the European Alpine Space.

## Materials and methods

### Study area

Our study area corresponds to the cooperation area of the European Alpine Space Programme, covering the European Alpine arc and its surrounding foothills, lowlands, and low-mountain ranges. Between 2014 and 2018, the region had, on average, 67.5 million inhabitants ^29^, living in Italian (IT, 34.5%), French (FR, 21.7%), German (DE, 15.5%), Austrian (AT, 12.9%), Swiss (CH, 12.3%), and Slovenian (SI, 3.1%) Alpine Space territories. At the same time, an average of almost 500 million overnight stays have been registered per year ^29^, which corresponds to a total of 1.4 million resident equivalents. We added these equivalents to the population data of the corresponding NUTS 2 regions for the purpose of our study. Liechtenstein (0.06% of the Alpine population) was not considered, as essential input data were not available. The study area is topographically diverse, with the highest elevation at 4,810 m a.s.l. in the Western European Alps and 0 m a.s.l. close to the Mediterranean Sea. From its margins and lower elevations to the Central Alps, the study area spans from warm-temperate climates with warm to hot summers to boreal to alpine climates ^53^.

In the study area, depending on the local climatic and topographic conditions, a mixture of grassland use and arable land is the traditional basis of agriculture. With industrialization and globalization since the nineteenth century, parts of these cultural landscapes have disappeared due to abandonment or urban sprawl ^26^. Today, 35% of the land surface area is used as agricultural land (ca. 135,000 km^2^), which is composed of arable land (46%), permanent cultures such as fruit trees and vineyards (5%), and grasslands ^29^. Strong regional differences apply for farming activities: arable land and permanent cultures are mainly concentrated in the lower valley bottoms and the peri-Alpine belt, with permanent cultures predominantly existing in climatically more favourable zones. Grasslands can mainly be found in climatically or topographically less favourable areas, such as higher valleys and steeper slopes, in the subalpine-alpine zone (summer pastures) or in the northern peri-Alpine belt ^45^.

### Methods

**Figure 4:**
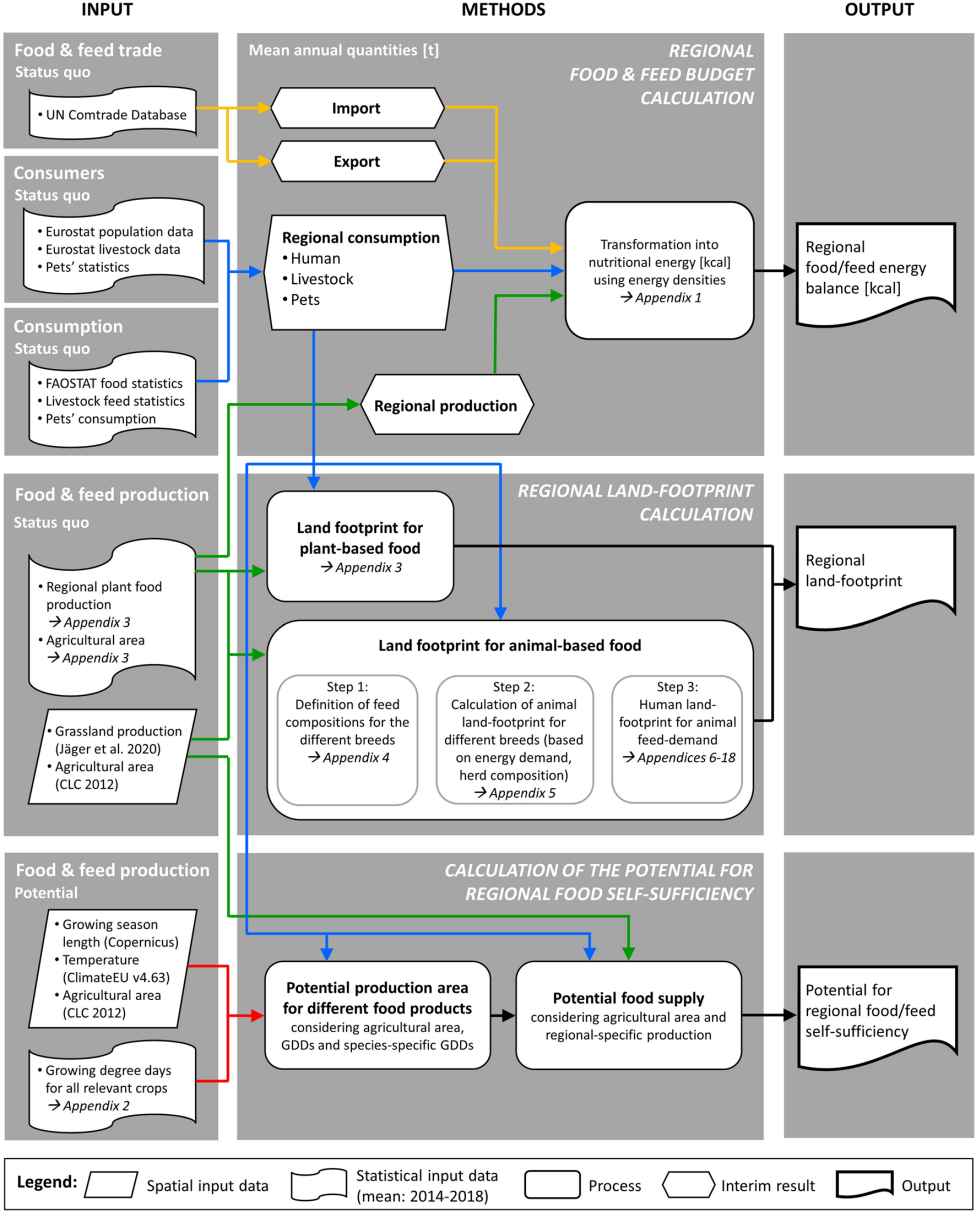
Approaches for modelling food and feed energy budgets, the land footprint, and the potential for food self-sufficiency at the regional level. Data sources and references to data tables in the Appendix are provided where necessary.

Our approach consisted of three steps, which are described more in detail in the following sub-chapters (cf. Fig. 4): (4.2.1) we calculated the current regional food and feed energy balance, (4.2.2) we derived the regional land footprint per capita on the basis of the currently consumed food and feed, and (4.2.3) we modelled the potential for regional food and feed self-sufficiency. For our modelling at the regional level, we used NUTS 2 regions as delimitations.

#### Current regional food and feed balance

To quantify the production, import, export and consumption components of the current food system, we calculated food balances similar to the FAOSTAT national food balance sheets ^28^ but for each NUTS 2 region of our study area and using regional data (where available) on food and feed production, trade, and consumption. To ensure stable datasets and account for annual variation in the data, for 2014–2018, we averaged regional data on food production (mainly from national and regional statistical offices, cf. full list of sources in Appendix 3), international trade ^54^, food consumption ^55^, livestock feed consumption (cf. sources in Appendices 4–18), and pet consumption (cf. full list of sources in Fig. 4). As a next step, we calculated a food balance at the national level for each country in our study area to assess the quality of the baseline data and thus ensure the comparability of the different datasets. We then applied the results to our regional study area by downscaling the consumption and international trade data using the national per capita averages and population and tourist data ^29^.

For each region, the following energy balance calculation was performed:

$$\sum (P,I){-} \, \sum (E, \, {\mathbf{HC}},{\mathbf{LC}}, \, {\mathbf{PC}}) \approx {\mathbf{0}} \, \pm \, {\mathbf{R}}$$

P = total production; I = total imports; E = total exports; HC = human consumption; LC = livestock consumption; PC = pet consumption; R = not attributable/remainder.

Production data for 148 food products, trade data aggregated to 56 product groups and human consumption data for 48 food product groups were included in the calculation. Furthermore, we calculated the annual feed energy demand per breed (cf. sources in Appendix 4) taking into account the mean herd composition (cf. sources in Appendix 5) and the maintenance and performance energy demand (cf. Sources in Appendices 6–18). For food and feed production, we were able to use data that were mostly available at the regional level. We did not take into account changes in regional and national food stocks in the balance calculations, as increasingly efficient food systems have generally led to a reduction in food stocks ^56^. To make production, trade and consumption data for humans, livestock and pets comparable, we calculated the nutrient energy by converting weight (t) into calories using the specific energy densities of the food commodities (cf. Appendix 1). Due to the different energy densities of different foods and feeds, total weight and calorie content can vary considerably. Since production, trade and consumption data were obtained from different statistics, complete consistency in the food and feed energy balance was not always possible. In these cases, the remainder was declared not attributable.

#### Current regional consumption land footprint

To calculate the per capita land footprint (m^2^ yr^-1^), we used the food consumption data calculated in the previous step, excluding food waste per region. Food waste at the consumer level was calculated by downscaling national data ^57^ per capita for the waste categories ‘Retail and other distribution of food’, ‘Restaurants and food services’ and ‘Households’ and relating it to consumption data. The share of food waste varies considerably between the Alpine regions ranging from 8.6% (SI) to 21.3% (CH, cf. Appendix 20). We then determined how much permanent grassland, arable land, and permanent cropland is required to regionally produce the food currently consumed in the study area, taking into account the regional growth potentials based on regional production statistics (cf. Appendix 3). Furthermore, we summarized the required areas for the production of animal-based foods (meat, milk, fish and eggs) based on the feed quantities, on the compositions of livestock species (Appendix 4) and herd composition (Appendix 5), using feeding guide values based on scientific results, experimental results and practical experiences (see Appendix 4) . For milk (Appendix 6) and egg production (Appendix 7), we added the performance-energy demand to the maintenance-energy demand. The calculation of the land consumption in the regions for pigs and fish was complicated by the fact that their feed composition also consists of animal products. A wide variety of dairy products are used in pig fattening ^58^, which we covered in our study with domestic dairy products. In fish farming (especially for predatory fish such as trout, pike, or char), a considerable proportion of the feed consists of fish meal and other fish derivatives, which currently mainly consist of sea catches ^59,60^. Here, we assumed that a high feed proportion could be substituted by vegetable protein products, and the remaining proportion could be covered by meat or fish derivatives of predominantly herbivorous freshwater fish.

#### Regional potential for food self-sufficiency

To model the potential for regional food production, we first calculated the growing degree-days (GDDs) for all agriculturally used areas. The GDDs show the accumulated temperature available during the growing season ^61^. The GDDs were calculated each day as the mean temperature minus the base temperature ^62^. GDDs were accumulated by adding these daily growth degrees over the growing season length ^63^. Then, we collected the GDD reference values for all agricultural crops currently consumed (cf. Appendix 2), which ranged between 595 (spinach) and 4803 GDDs (citrus fruits). Using these reference values together with local mean production yields, due to different production conditions (cf. Appendix 3), we were able to calculate the theoretically necessary agricultural area within each GDD class and each region to meet the regional food demand. The demand for animal-based products was included by using the quantity of feed necessary for maintenance and performance energy. In a final step, we balanced the available and theoretically necessary agricultural GDD areas in each region, also considering potential cropping sequences in areas with long season lengths and high GDDs, resulting in up to three cropping sequences per year. The balance was achieved by subtracting the theoretically UAAs (cf. sources in Appendix 3) required within a given GDD class from the actually UAA available within each class; the resulting shortfalls and surpluses were merged by standardizing them to 1250 GDDs. We assumed that all current UAAs with a slope < 16° were suitable for mechanized arable farming, viticulture, and fruit production and that all remaining were suitable for grassland farming. This is a strict assumption, as we are aware that steeper areas are also suitable for arable farming and viticulture but require more time and effort. Thus, we were able to obtain the potential for food self-sufficiency in the European Alps based on local geographic conditions. To georeference the regional UAAs, we used Corine Land Cover agricultural land-use classes ^64^ and corrected them using the statistical UAA information ^29^.

### Supplementary Information

Supplementary Information.

## Data Availability

All data generated or analysed during this study were obtained from publicly available datasets or are included in this published article and its Supplementary Information files.
