# Supplementary material for: Regional food self-sufficiency potential in the European Alpine space
Source: Sci Rep. 2024 Apr 25;14:9527. doi: 10.1038/s41598-024-60010-z (PMC11045778; doi:10.1038/s41598-024-60010-z)
Supplement: Supplementary file 1 — Supplementary Information. [file 41598_2024_60010_MOESM1_ESM.pdf]

APPENDICES

Appendix 1: Energy densities (ED) of important food and feed products. Data are based on harvested or fresh weight, or dry weight for feed.
Data sources: www.bmi-rechner.net/energiedichte-tabelle.htm (food), www.lfl.bayern.de (animal feed)

Food products	ED (kcal g^{-1})	Food products	ED (kcal g^{-1})	Food products	ED (kcal g^{-1})
**Cereals**	**2.9**	**Permanent grassland products**	**2.2**	Raisins	3.2
Wheat	2.6	Permanent grassland (1-cut)	1.7	Bush Berries	0.5
Wheat - Common wheat	2.6	Permanent grassland (more cuts)	2.2	Currants	0.4
Wheat - Durum wheat	2.6	Litter meadows	1.7	Strawberries	0.4
Wheat – Spelt	2.7	**Vegetables**	**0.4**	Gooseberries	0.5
Rye & Maslin	2.6	Broccoli	0.3	Raspberries	0.3
Mixed grain	2.6	Chinese cabbage	0.1	Elderberries	0.4
Oats	3.5	Runner beans	0.3	Blueberries	0.4
Triticale	2.6	Green Peas	0.8	chokeberry	1.1
Barley	3.4	Cucumbers	0.1	Wine production	0.7
Corn	3.3	Beetle beans	0.9	Total wine	0.7
Millet (grain)	3.4	Cauliflower	0.3	Red wine grapes	0.8
Other cereals (excl. millet)	2.9	Carrots	0.3	White wine grapes	0.8
Other cereals (incl. sorghum)	2.9	Garlic	1.4	Citrus fruits	0.3
Sorghum	3.4	Tuber fennel	0.3	Olives	1.9
Other cereals (excl. sorghum)	2.8	Cabbage	0.3	Bergamot	0.5
Rice	3.3	Kohlrabi	0.3	Bitter Orange	0.5
**Pulses**	**2.0**	Brussels sprouts	0.3	Clementine	0.5
Field peas	0.8	Total cabbage	0.3	Melon	0.5
Field beans	1.3	Horseradish	0.6	Fig	0.6
Vetches	1.3	Eggplant	0.3	Kiwi	0.5
Peas other than field peas	0.8	Melon	0.3	**Beer, alcoholic beverages**	**0.4**
Kidney beans	3.0	Sweet peppers	0.4	**Oils**	**8.9**
Sweet lupines	3.2	Parsley green	0.2	Olive oil	8.9
Lentils, chickpeas, vetches	2.8	Parsley root	0.5	Coconut oil	9.0
Pulses (peas, beans, vetches)	1.9	Pepperoni	0.4	Cottonseed oil	8.8
Soybeans	3.3	Leeks	0.3	Oilseeds Oil	8.9
Oilcake (mainly soya-bean)	5.8	Radishes	0.2	Palm oil	8.8
Other dried pulses	2.0	Beer radish	0.2	Palm kernel oil	8.8
**Oleaginous fruits**	**4.8**	Rhubarb	0.2	Rape and mustard oil	9.0
Sunflowers	5.6	Beetroot	0.5	Rice oil	8.9
Canola	5.3	Salad	0.2	Sunflower seed oil	9.0
Pumpkin seeds	6.5	Chives	0.3	**Meat**	**1.4**
Poppy	4.9	Celery	0.3	Beef	1.2
Oil flax	4.3	Total asparagus	0.2	Pork	1.5
Other oil plants	2.4	Squash	0.3	Poultry meat	1.6
**Starchy tubers**	**1.3**	Spinach	0.3	Sheep and goat meat	1.9
Potatoes	0.8	Tomatoes	0.2	Horsemeat	1.0
Early potatoes	0.8	Corvette	0.2	Other meat	1.4
Late potatoes	0.8	Sweet corn	0.9	**Milk products**	**3.3**
Sugar Beet	2.9	Onions	0.3	Milk + fresh milk products	0.6
**Temporary forage products**	**2.3**	**Fruit production**	**0.5**	Cream	2.0
Fodder beet	2.9	Apples	0.5	Butter	7.4
Hops	2.5	Pears	0.5	Milk powder	5.0
Clover, alfalfa,	2.0	Nuts	6.6	Cheese	2.7
Temporary grasses and willows	2.4	Peach	0.5	Hard cheese	3.9
Silage and green corn	3.3	Apricots	0.4	Cream cheese	1.4
Grain harvested green	2.4	Plums	0.6	**Eggs**	**1.0**

Other green mass	2.4	Cherries	0.6	**Honey**	**3.1**
Straw	1.3	Sour cherries	0.4	**Fish and seafood**	**0.7**
Energy crops	2.4	Grapes	0.7		

Appendix 2: Needed GDD until maturity for all relevant crops in the Alpine Space region, including literature sources. Values are standardized to a base temperature of 1°C .

Crops	GDD (1°C)	Source
**Cereals**		
Wheat	1966	1
Common wheat	1966	1
Durum wheat	1608	2, 1
Spelt	2100	3
Rye	1877	1
Oats	1618	2, 1
Triticale	1938	1
Barley	1478	2, 1
Grain maize	2700	1
Millet	2438	4
Sorghum	2113	5
Rice	3730	6
**Leguminous plants**		
Pea	929	1, 7
Broad and field bean	1082	1, 7
Vetches	847	7
Peas	1082	2, 7
Kidney beans	963	7
Sweet lupines	1469	8
Lentils, Chick-peas	1775	2, 1
Broad beans, French beans	1164	7
**Oleaginous fruits**		
Soya bean	1138	7
Sunflower	1876	2
Rape	1495	2
Pumpkin seeds	1838	7
Poppy	1876	2
Flax	1876	2
Other (sesame, hemp)	1683	2
**Tuber fruits**		
Early potatoes	1309	7
Potatoes	1373	1, 7
Sugar beet	1482	1, 7
**Total temporary forage**		
Fodder beet	1393	2, 7
Hops	2900	9
Lucerne	1183	7
Gramineae	1183	7
Silage and green corn	2700	1
Forage barley	1183	7
Other mixtures	1183	7
Hemp (straw)	1183	7
**Permanent grassland**		
Pasture	400	10
Permanent hay meadow	708	10
Poor grassland	400	10
**Vegetables**		
**Vegetables (continued)**		
Carrots and parsnips	2394	7, 11
Garlic and shallot	3458	7, 11
Tuber fennel	1107	7, 11
Cabbage	1569	7, 11
Kohlrabi	971	7, 11
Brussels sprouts	1940	7, 11
Total cabbage	2367	7, 11
Horseradish	1729	7, 11
Chard	1190	7, 11
Egg-plant	2258	7, 11
Melon	1731	7, 11
Pepper	2860	7, 11
Parsley root	1064	7, 11
Pfefferoni	2860	7, 11
Leeks	1428	7, 11
Radishes	714	7, 11
Beer radish	1729	7, 11
Rhubarb	1806	7, 11
Beetroot and leaf beet	931	7, 11
Lettuce	952	7, 11
Chive	1064	7, 11
Celery	1428	7, 11
Asparagus	1806	7, 11
Pumpkin	2634	7, 11
Spinach	595	7, 11
Tomato	1806	7, 11
Watermelon	3311	7, 11
Zucchini	2559	7, 11
Sweet corn	2860	7, 11
Onions	1666	7, 11
**Fruit production**		
Apple	2962	1
Apple	2962	1
Walnut	2930	12
Peach	4021	13
Apricot	2926	13
Plum	3047	1
Cherry	2161	1
Sour cherries	2749	1
Grapes	3290	14
Kiwi	2926	13
**Bush berries**		
Red currant	2069	15
Strawberries	909	16
Gooseberries	2069	15
Raspberries	2069	15
Elderberries	2069	15

Artichoke	2860	[7, 11]	Blueberries	2069	[15]
Broccoli	1303	[7, 11]	Chokeberry	4021	[13]
Chinese cabbage	904	[7, 11]	**Wine production**		
Beans	1656	[7, 11]	Red wine grapes	3355	[14]
Green peas	1190	[7, 11]	White wine grapes	3210	[14]
Cucumbers	1430	[7, 11]	**Citrus fruit**	**4803**	**[17]**
Beetle beans	1656	[7, 11]	**Olives**	**4100**	**[18]**
Cauliflower and broccoli	2633	[7, 11]			

Appendix 3: Regional average areal yield (t ha^{-1}) and current used area per crop (ha) (data sources: Austria: [19], Germany: [20, 21, 22], Italy: [23], Switzerland: [24, 25], France: [26,27,28,29], Liechtenstein: [30], Slovenia: [31]; for permanent grassland: [32])

	Austria		Germany		Italy		Switzerland		France		Liechtenstein		Slovenia	
	Yield (t ha^{-1})	Area (ha)	Yield (t ha^{-1})	Area (ha)	Yield (t ha^{-1})	Area (ha)	Yield (t ha^{-1})	Area (ha)	Yield (t ha^{-1})	Area (ha)	Yield (t ha^{-1})	Area (ha)	Yield (t ha^{-1})	Area (ha)
**Cereals**														
Wheat			**4.6**	263731.2			**5.8**	83492.6			**5.8**	98.9	**5.0**	30559.4
Common wheat	**5.6**	265449.5			**5.9**	240786.5			**6.3**	234811.8	**5.4**	21.9		
Durum wheat	**7.3**	21780.8			**5.6**	39062.0								
Spelt	**2.5**	13372.9					**3.0**	4190.0						
Rye	**4.7**	40063.5	**3.2**	13220.1	**3.7**	1212.5	**4.3**	1894.7	**4.4**	4856.6	**4.3**	2.8	**3.7**	1135.9
Oats							**3.7**	380.5		0.0				
Triticale	**4.1**	22801.3	**1.9**	27054.9	**4.0**	949.0	**3.9**	1654.1	**3.5**	6304.8			**3.1**	1453.6
Barley			**3.2**	23949.2			**5.3**	8571.9	**5.1**	27435.0	**5.3**	8.4	**4.4**	4295.8
Grain maize	**5.5**	142591.5	**3.2**	165932.6	**5.5**	59911.5	**5.3**	28065.4	**5.6**	90124.2	**5.3**	48.6	**4.5**	18958.6
Millet	**19.9**	203935.0	**15.2**	64927.3	**10.6**	516982.0	**10.1**	15694.2	**10.1**	258841.4	**10.1**	25.3	**8.2**	38385.8
Sorghum	**7.2**	2910.2			**6.0**	9187.5	**3.8**	1161.3	**5.9**	7906.4				
Other cereals	**3.5**	21147.1			**4.3**	3528.5			**3.6**	5618.0	**3.5**	9.3	**1.3**	**3094.9**
Rice					**6.8**	218509.0			**5.6**	11192.6				
**Leguminous plants**														
Pea	**2.9**	7103.5	**3.0**	4563.0	**3.4**	2305.5	**2.3**	3965.7	**2.7**	4235.8	**2.3**	4.0	**2.5**	455.4
Broad and field bean	**2.0**	9441.7	**3.3**	1450.7	**3.5**	117.0	**2.4**	638.3	**2.2**	1233.4			**1.7**	439.7
Vetches					**2.0**	2526.5								
Peas					**3.8**	1053.5			**2.0**	477.0				
Kidney beans					**6.8**	2620.0								
Sweet lupines	**1.7**	164.0					**2.0**	98.8	**2.5**	69.0				
Lentils, Chick-peas	**1.5**	2322.9			**2.5**	219.5			**1.3**	111.0				
Other beans	**2.5**	3295.0	**12.4**	12.6	**6.1**	2004.0	**6.4**	649.7						
**Oleaginous fruits**														
Soya bean	**2.3**	56522.1			**3.7**	263520.5	**2.8**	1567.1	**2.9**	30015.6	**2.8**	2.4	**2.8**	1097.0
Sunflower	**2.8**	20262.5	**2.6**	244.3	**3.8**	8554.0	**2.6**	4509.2	**2.1**	29675.2			**2.3**	280.8
Rape	**7.3**	40224.0	**2.1**	62622.4	**2.8**	6982.5	**3.0**	22166.7	**3.5**	59281.6	**3.1**	11.9	**2.9**	4280.6
Pumpkin seeds	**0.5**	32229.9					**0.6**	53.6					**0.6**	4694.0
Poppy	**0.7**	2446.2												
Flax	**0.3**	3774.6			**2.9**	5.5	**1.3**	154.9						
Other (sesame, hemp)					**10.0**	1.0			**1.8**	586.4			**0.8**	249.3
**Tuber fruits**														
Early potatoes	**27.1**	12780.5	**33.4**	25191.3	**12.1**	343.0	**32.5**	11075.0	**30.3**	3083.6			**25.0**	3390.8
Potatoes	**36.9**	9163.9			**32.2**	6758.5					**36.9**	76.1		
Sugar beet			**68.1**	14899.9	**62.0**	13811.5	**67.6**	19530.1	**84.6**	6541.2	**68.2**	24.0		
**Temporary forage**													**25.1**	55478.3
Fodder beet	**68.2**	128.7	**60.0**	3643.9			**55.0**	556.8			**55.0**	3.6		
Hops	**1.7**	248.8	**1.7**	9.2			**1.7**	18.0					**1.5**	1403.7
Lucerne	**7.4**	78140.7	**0.0**	32001.3	**32.6**	118598.0			**20.1**	46826.5			**6.9**	26766.7
Gramineae			**12.0**	99139.6	**27.9**	10613.5	**6.9**	127294.4			**6.9**	477.6		
Silage and green corn	**45.8**	85542.5	**36.4**	203385.8	**51.5**	231416.5	**44.9**	46649.6	**10.8**	94251.6	**44.9**	397.2	**44.1**	27308.0
Forage barley	**20.5**	1049.4			**24.1**	1334.5								
Other mixtures	**28.9**	17396.7	**29.2**	4915.2	**30.2**	104709.0	**29.8**	36.5	**9.6**	6540.3				
Hemp (straw)	**3.9**	1263.6			**9.2**	273.5	**4.2**	481.7						
**Permanent grassland**	**6.3**	1878098.9	**9.1**	956961.3	**5.5**	997475.2	**5.8**	1301970.7	**6.4**	2080966.6	**3.8**	4549.0	**6.9**	474319.0
**Vegetables**											**38.8**	138.4		
Artichoke														
Broccoli	**15.4**	132.7	**20.7**	34.3	**26.8**	726.0	**17.7**	605.6					**15.3**	88.9
Chinese cabbage	**57.8**	382.8	**43.3**	39.8	**24.8**	265.0								
Beans	**13.5**	414.5							**8.2**	434.5				
Green peas	**5.3**	1895.4			**6.1**	1963.0			**5.7**	104.0			**3.4**	57.9
Cucumbers	**120.0**	373.7	**115.2**	17.0	**29.4**	73.0	**115.2**	171.0					**19.9**	135.0
Beetle beans	**0.6**	686.3			**5.5**	41.0			**2.0**	36.5				
Cauliflower and broccoli	**29.8**	127.8	**32.8**	81.6			**27.0**	2978.9						
Carrots and parsnips	**53.6**	1767.4	**38.7**	763.1	**46.5**	593.0	**55.2**	3009.9	**37.1**	715.0			**19.7**	192.3
Garlic and shallot	**4.3**	185.5			**8.7**	555.0			**9.0**	670.5			**6.3**	122.9
Tuber fennel	**15.3**	37.1			**23.1**	167.5	**20.4**	100.4						
Cabbage	**47.8**	65.5			**35.8**	323.0	**40.1**	491.0	**45.9**	1523.5			**32.5**	608.2
Kohlrabi	**28.4**	108.1	**35.4**	44.3	**35.4**	140.0								
Brussels sprouts	**10.3**	25.7												
Total cabbage	**65.7**	746.9	**34.3**	857.8	**27.6**	826.0	**58.0**	144.6						
Horseradish														
Chard														
Egg-plant	**92.2**	8.6			**34.9**	290.5			**52.9**	179.0				
Melon	**35.4**	18.7			**29.5**	3746.5								

Pepper	**26.0**	154.2	**61.8**	2.9	**25.2**	452.0	**61.3**	97.9					**25.7**	143.6
Parsley	**23.9**	69.8			**27.6**	45.5								
Parsley root	**32.8**	104.2												
Pfefferoni	**26.0**	16.8												
Leeks	**39.6**	140.2	**44.5**	24.0										
Radishes	**17.9**	293.4	**22.9**	20.2	**16.2**	14.0	**17.5**	859.3						
Beer radish	**42.5**	49.3	**43.5**	80.7					**15.8**	765.5				
Rhubarb	**29.0**	40.4	**30.4**	9.3			**29.4**	106.1			**29.4**	1.2		
Beetroot and leaf beet	**57.4**	139.8	**56.8**	91.0	**20.1**	203.5							**22.2**	129.1
Lettuce	**29.0**	1606.0	**2.4**	2025.0	**17.7**	9226.0	**29.2**	1233.8	**27.5**	5240.0			**15.0**	1124.9
Chives	**11.7**	231.0												
Celery	**43.3**	317.7	**49.7**	122.0	**34.8**	133.5	**43.5**	689.3	**37.0**	116.5				
Asparagus	**0.0**	736.5	**3.5**	2339.6	**4.8**	2094.0	**4.2**	425.4	**3.3**	894.5	**4.2**	1.8	**3.8**	53.0
Pumpkin	**29.2**	513.3	**15.7**	493.9			**32.8**	101.5						
Spinach	**55.0**	224.5	**18.7**	18.3	**12.9**	622.0			**16.3**	535.5			**11.2**	24.7
Tomato	**51.6**	185.9			**61.1**	**12242.0**	**292.8**	202.4	**125.9**	1814.0			**36.1**	197.7
Watermelon														
Courgette	**37.4**	147.8	**36.2**	7768.8	**21.0**	3790.0	**40.8**	590.9					**23.4**	120.1
Sweet corn	**16.3**	765.6	**5.3**	39.5										
Onions	**35.9**	3384.7	**35.9**	111.3	**35.9**	3057.5	**50.1**	2050.1	**26.7**	945.0			**21.3**	354.4
**Fruit production**							**0.0**	444.2						
Apple	**38.0**	6691.0	**38.0**	16892.6	**53.3**	40481.5	**37.6**	4522.1	**38.3**	13825.0	**37.6**	1.1	**25.3**	2550.3
Pear	**123.4**	458.2	57.7	**918.3**	**27.2**	5161.0	**105.6**	775.4	**25.0**	3300.0	**105.6**	0.5	**15.9**	206.9
Walnut	**24.8**	147.7							**1.8**	9413.5			**2.3**	225.2
Peach	**13.8**	168.6			**25.5**	7899.0			**25.5**	4114.5			**14.5**	359.1
Apricot	**9.8**	781.4			**12.3**	1423.5	**10.0**	475.3	**14.9**	8171.0			**7.5**	54.2
Plum	**82.8**	188.6	**41.9**	1776.4	**21.6**	1586.0	**62.2**	5.2	**13.3**	1062.0	**62.2**	2.1	**9.3**	36.2
Cherry	**29.7**	229.6	**14.5**	2724.7	**5.1**	2958.5	**25.1**	9.8	**5.9**	5442.5			**6.7**	155.9
Sour cherries	**30.9**	22.8	**18.2**	196.1									**5.6**	9.8
Grapes					**10.0**	178.5							**6.6**	16014.7
Kiwi					**22.6**	8815.0			**16.1**	380.5				
Citrus fruit					**9.5**	53.0			**11.2**	16.5				
Olives					**2.2**	23392.0			**1.5**	11261.0			**1.7**	1043.4
**Bush berries**			**29.1**	252.0							**8.4**	0.8		
Red currant	**12.9**	259.3	**35.3**	503.9	**6.7**	100.5	**12.2**	65.8	**3.4**	354.0			**4.5**	122.4
Strawberries	**10.4**	1152.9	**12.6**	2768.8	**19.5**	677.5	**10.7**	475.1			**10.7**	2.0	**18.1**	101.9
Gooseberries					**3.3**	5.0	**4.0**	8.2	**5.5**	58.0				
Rouspberries	**5.2**	159.7	**18.7**	262.5	**7.3**	278.0	**5.2**	305.4	**7.3**	254.0				
Holunderbeeren	**2.2**	1326.7	**2.2**	35.7			**6.6**	38.6						
Blueberries	**6.1**	154.0	**2.0**	85.9			**6.8**	131.0						
Chokeberry	**3.1**	484.4												
**Wine production**														
Wine grapes, mixed	**49.5**	46208.1	**49.5**	11384.3	**12.8**	179555.0	**5.1**	13276.1	**5.3**	150587.5	**5.1**	8.2		
Red wine grapes	**47.3**	15705.7												
White wine grapes	**50.7**	30502.4												
Wine grapes, mixed														

Appendix 4: Proportions of feed compositions for the different breeds, including information on data sources and average daily feed quantity per species (weighted according to herd composition, cf. Appendix 5) and per milk produced. Only feed contents > 0.1% are listed.

	**DM of harvest mass (%)**	**Cattle**	**Horse**	**Sheep**	**Goat**	**Pig**	**Chicken**	**Goose**	**Duck/ Guinea fowl**	**Turkey**
		(% of DM)								
Grass/ hay	0.86	40.2	47.8	60.4	60.4	0.7	0.00	0,0	0,0	0,0
Cereal grass/alfalfa	0.86	12.4	0.0	0.0	0.0	1.1	0.1	0,1	0,1	0,1
Straw	0.86	2.6	17.4	0.0	0.0	0.0	0.0	0,0	0,0	0,0
Maize	0.9	19.1	4.6	8.0	8.0	11.7	18.6	18,6	18,6	18,6
Wheat	0.88	1.0	1.7	7.3	7.3	11.5	31.1	31,1	31,1	31,1
Barley	0.88	2.1	2.3	10.7	10.7	9.6	2.1	2,1	2,1	2,1
Triticale	0.88	0.0	0.0	0.0	0.0	6.5	3.0	3,0	3,0	3,0
Oats	0.88	1.9	20.0	3.1	3.1	2.3	0.1	0,1	0,1	0,1
Millet	0.88	0.0	0.0	0.0	0.0	0.0	0.2	0,2	0,2	0,2
Sesame	0.88	0.0	0.0	0.0	0.0	0.0	0.1	0,1	0,1	0,1
Soya	0.89	6.9	1.7	0.3	0.3	5.2	16.4	16,4	16,4	16,4
Rapeseed	0.88	3.7	0.0	0.0	0.0	3.2	3.7	3,7	3,7	3,7
Sunflowers	0.88	0.0	0.0	0.0	0.0	1.5	3.9	3,9	3,9	3,9
Lucerne meal	0.89	0.0	0.0	0.0	0.0	0.7	2.6	2,6	2,6	2,6
Peas	0.88	2.1	0.0	0.0	0.0	2.4	2.3	2,3	2,3	2,3
Broad beans	0.88	2.1	0.0	8.3	8.3	1.9	0.3	0,3	0,3	0,3
Linseed	0.90	0.0	1.2	0.0	0.0	2.4	0.3	0,3	0,3	0,3
Carrots	0.15	0.0	1.5	0.0	0.0	0.0	0.0	0,0	0,0	0,0
Brewer's yeast	0.90	0.9	0.6	0.0	0.0	2.9	0.0	0,0	0,0	0,0
Sugar beet	0.27	3.8	1.2	0.0	0.0	8.1	0.0	0,0	0,0	0,0
Potato	0.18	0.5	0.0	0.0	0.0	10.0	0.0	0,0	0,0	0,0
Manioc	0.88	0.0	0.0	0.0	0.0	3.1	0.0	0,0	0,0	0,0
Rye	0.88	0.0	0.0	0.0	0.0	9.4	0.0	0,0	0,0	0,0
Sweet lupine	0.88	0.0	0.0	0.0	0.0	1.6	0.0	0,0	0,0	0,0
Milk products	0.13	0.0	0.0	0.0	0.0	3.8	0.0	0,0	0,0	0,0
Fodder oil	1.00	0.0	0.0	0.0	0.0	0.3	3.1	3,1	3,1	3,1
Feeding lime	1.00	0.7	0.0	1.7	1.7	0.0	9.8	9,8	9,8	9,8
Animal salt	1.00	0.0	0.0	0.2	0.2	0.0	0.1	0,1	0,1	0,1
Artificial additives	1.00	0.0	0.0	0.0	0.0	0.0	2.3	2,3	2,3	2,3
	**Source:**	33, 34, 35	36	33	33	37	38, 39, 40	38, 40	38, 40	38, 40
**DM consumption per adult** (kg d^{-1})		12.54	9.90	2.21	2.21	1.8	0.10	0.14	0.12	0.20
**Milk yield consumption** (kg l^{-1} d^{-1})		0.174								

Appendix 5: Used herd composition, energy demand and average feed dry mass per individual and herd class (data sources: cattle: [41, 42, 43]; horse: [44]; sheep: 41; goat: [41]; pig: [45]; chicken: [46, 47, 48]; goose: [46, 48]; duck/ Guinea fowl: [46, 48]; turkey: [46, 48])

Herd class	% of herd	Energy demand (MJ ME d^{-1})	Feed DM (g d^{-1})	Herd class	% of herd	Energy demand (MJ ME d^{-1})	Feed DM (g d^{-1}
**Cattle**				**Pig**			
Milk cow (30 l d^{-1})	63.1	213.6	12.5	Porker	85.6	47.3	1.51
Calves	6.9	47.5	3.9	Breeding sow	14.1	56.4	1.80
Breeding cattle	27.8	109.2	8.9	Breeding boars	0.3	56.4	1.80
Bull/Stores	2.2	160.6	13.2	*Mean demand:*		*48.6*	*1.55*
Mean demand:		*133.8*	*11.0*	**Chicken (places)**			
**Horse**				Laying hen	47.0	2.1	0.10
Mare (600 kg)	50.0	161.5	9.9	Parent animal	0.5	2.1	0.10
Foals	15.0	120.2	2.3	Chicken for fattening	52.5	1.4	0.07
Young horse	20.0	129.3	3.9	*Mean demand:*		*1.7*	*0.08*
Stallion (600 kg)	15.0	147.3	9.0	**Turkey**			
Mean demand:		*121.5*	*7.4*	Fattening cock	45.0	4.5	0.46
**Sheep**				Fattening hen	45.0	2.8	0.28
Ewe (70kg)	60.0	27.1	2.2	Parent animal	10.0	4.6	0.47
Young sheep	20.0	21.7	1.8	*Mean demand:*		*3.7*	*0.38*
Fattening lamb	15.0	23.2	1.9	**Goose**			
Ram	5.0	27,1	2.2	Fattening goose	90.0	2.6	0.07
Mean demand:		*25,4*	*2.1*	Parent animal	10.0	4.4	0.14
**Goat**	60	27.6		*Mean demand:*		*2.8*	*0.09*
Milk goat	60	27.6	2.2	**Duck, Guinea fowl**			
Goat lamb	15	12.6	1.0	Fattening duck, fowl	98.0	0.8	0.07
Young goat	20	17.2	1.4	Parent animal	2.0	1.4	0.12
Billy goat	5	25.8	2.1	*Mean demand:*		*0.9*	*0.07*
Mean demand		*23.2*	*1.9*				

Appendix 6: Current milk production: feed demand and required production area per crop and country, and average milk production per area. The data are based on the data in Appendices 3 (areal yield), 4 (feed composition) and 5 (energy demand), as well as the data on dairy cows per country (data sources: Austria: [49], Germany: [50], Italy: [52], Switzerland: [51], France: [53], Liechtenstein: [54], Slovenia: [55]).

	AT		DE		CH		IT		FR		LI		SI	
	Demand (10^3 t a^{-1})	Area (10^3 ha)	Demand (10^3 t a^{-1})	Area (10^3 ha)	Demand (10^3 t a^{-1})	Area (10^3 ha)	Demand (10^3 t a^{-1})	Area (10^3 ha)	Demand (10^3 t a^{-1})	Area (10^3 ha)	Demand (10^3 t a^{-1})	Area (10^3 ha)	Demand (10^3 t a^{-1})	Area (10^3 ha)
Grass/ hay	2808.0	593.4	3700.1	517.5	2351.0	492.9	5590.0	1000,0	2757,9	427,6	9,3	2,5	732,0	137,3
Alfalfa	868.0	57.6	1143.7	64.3	726.7	40.9	1727.9	75,2	852,5	46,8	2,9	0,2	226,3	12,7
Straw	181.3	6.1	238.9	8.0	151.8	5.1	360.9	12,1	178,0	6,0	0,6	0,0	47,3	1,6
Maize	1332.3	29.6	1755.5	39.1	1115.4	24.8	2652.2	48,5	1308,5	29,1	4,4	0,1	347,3	7,9
Wheat	68.1	11.8	89.7	10.9	57.0	9.9	135.5	22,2	66,9	11,0	0,2	0,0	17,7	3,5
Barley	146.1	27.8	192.5	27.7	122.3	23.2	290.8	62,4	143,5	24,3	0,5	0,1	38,1	8,4
Oats	284.7	73.6	173.8	34.2	110.4	28.5	262.5	79,5	129,5	29,1	0,4	0,1	34,4	11,2
Soya	1039.5	364.7	634.6	217.1	403.2	141.5	958.8	274,6	473,0	174,6	1,6	0,6	125,5	45,0
Rapeseed	565.3	180.1	345.1	76.7	219.3	73.6	521.4	173,0	257,2	75,2	0,9	0,3	68,3	23,9
Peas	324.1	140.1	197.9	55.3	125.7	54.4	298.9	86,2	147,5	48,2	0,5	0,2	39,1	15,7
Broad beans	324.1	134.3	197.9	55.3	125.7	52.1	298.9	120,3	147,5	57,7	0,5	0,2	39,1	22,6
Linseed	4.8	3.8	2.9	2.3	1.9	1.5	4.4	3,5	2,2	1,7	0,0	0,0	0,6	0,5
Brewer's yeast	130.2	74.8	79.5	45.6	50.5	29.0	120.0	71,5	59,2	35,3	0,2	0,1	15,7	10,5
Sugar beet	567.7	8.2	346.6	3.8	220.2	3.3	523.6	7,7	258,3	2,8	0,9	0,0	68,6	0,9
Potato	68.3	2.1	41.7	1.5	26.5	0.8	63.0	2,6	31,1	0,7	0,1	0,0	8,2	0,3
Sweet lupine	5.4	2.7	3.3	1.6	2.1	1.1	5.0	2,4	2,4	1,1	0,0	0,0	0,6	0,3
Sum of area (ha)	1710850.3		1160916.6		982462.6		2042067.8		971323.7		4421.1		302364.7	
Milk units (t ha^{-1})	6.2		12.1		7.4		11.3		10.3		7.4		8.5	

Appendix 7: Current egg production: feed demand and required production area per crop and country, and average egg production per area. The data are based on the data in Appendices 3 (areal yield), 4 (feed composition) and 5 (energy demand), and on the data on laying hens per country (cf. livestock data source in Appendix 6).

	**AT**		**DE**		**CH**		**IT**		**FR**		**LI**		**SI**	
	Demand (10^3 t a^{-1})	Area (10^3 ha)	Demand (10^3 t a^{-1})	Area (10^3 ha)	Demand (10^3 t a^{-1})	Area (10^3 ha)	Demand (10^3 t a^{-1})	Area (10^3 ha)	Demand (10^3 t a^{-1})	Area (10^3 ha)	Demand (10^3 t a^{-1})	Area (10^3 ha)	Demand (10^3 t a^{-1})	Area (10^3 ha)
Alfalfa	0.1	0.0	1.2	0.1	0.2	0.0	0.7	0,0	1,6	0,1	0,0	0,0	0,1	0,0
Maize	36.9	3.7	301.9	28.0	37.5	3.7	163.4	15,1	394,9	43,1	0,0	0,0	20,2	2,5
Wheat	61.5	10.7	502.8	60.9	62.4	10.9	272.2	44,6	657,7	107,8	0,1	0,0	33,7	6,7
Barley	4.2	0.8	34.1	4.9	4.2	0.8	18.5	4,0	44,7	7,6	0,0	0,0	2,3	0,5
Triticale	5.9	1.1	47.8	6.6	5.9	1.1	25.9	4,7	62,6	11,4	0,0	0,0	3,2	0,7
Oats	0.2	0.0	1.5	0.3	0.2	0.0	0.8	0,2	1,9	0,4	0,0	0,0	0,1	0,0
Millet	0.3	0.1	2.5	0.8	0.3	0.1	1.3	0,4	3,2	1,1	0,0	0,0	0,2	0,1
Sesame	0.2	0.2	2.0	1.6	0.2	0.2	1.1	0,9	2,6	2,2	0,0	0,0	0,1	0,1
Soya	32.5	11.4	265.6	90.9	33.0	11.6	143.8	41,2	347,4	128,2	0,0	0,0	17,8	6,4
Rapeseed	7.4	2.4	60.3	13.4	7.5	2.5	32.7	10,8	78,9	23,1	0,0	0,0	4,0	1,4
Sunflowers	7.7	3.0	62.7	18.3	7.8	3.0	33.9	9,5	82,0	33,2	0,0	0,0	4,2	1,8
Lucerne meal	5.1	0.7	42.0	6.1	5.2	0.8	22.7	3,3	55,0	7,9	0,0	0,0	2,8	0,4
Peas	4.5	1.9	36.7	10.2	4.6	2.0	19.8	5,7	48,0	15,7	0,0	0,0	2,5	1,0
Broad beans	0.6	0.2	4.7	1.3	0.6	0.2	2.6	1,0	6,2	2,4	0,0	0,0	0,3	0,2
Linseed	0.6	0.4	4.5	3.6	0.6	0.4	2.4	1,9	5,9	4,7	0,0	0,0	0,3	0,2
Fodder oil	6.2	7.3	50.6	44.6	6.3	7.6	27.4	30,8	66,2	92,7	0,0	0,0	3,4	3,4
Sum of area (ha)	44031.1		291689.5		44988.8		174272.2		481565.2		43.4		25551.3	
Eggs without shell (t ha^{-1})	1.13		1.39		1.12		1.26		1.10		1.13		1.07	

Appendix 8: Current beef production: feed demand and required production area per crop and country, as well as meat production. Area calculations are based on the number of herds excluding dairy cows, as these are already included in milk production (cf. Appendix 6).
The data are based on the data in Appendices 3 (areal yield), 4 (feed composition) and 5 (energy demand) and the data on cattle per country (cf. data source for livestock in Appendix 6). The calculation of meat yield is based on slaughter information (source: www.ama.at/).

	**AT**		**DE**		**CH**		**IT**		**FR**		**LI**		**SI**	
	Demand (10^3 t a^{-1})	Area (10^3 ha)	Demand (10^3 t a^{-1})	Area (10^3 ha)	Demand (10^3 t a^{-1})	Area (10^3 ha)	Demand (10^3 t a^{-1})	Area (10^3 ha)	Demand (10^3 t a^{-1})	Area (10^3 ha)	Demand (10^3 t a^{-1})	Area (10^3 ha)	Demand (10^3 t a^{-1})	Area (10^3 ha)
Grass/ hay	786.8	138.2	1031.8	141.2	701.6	167.2	1506.5	540.9	781,7	150,2	2,7	0,6	209,9	27,8
Alfalfa	243.2	16.1	318.9	17.9	216.9	12.2	465.7	20.3	241,6	13,3	0,8	0,0	64,9	3,6
Straw	50.8	1.7	66.6	2.2	45.3	1.5	97.3	3.3	50,5	1,7	0,2	0,0	13,6	0,5
Maize	373.3	8.3	489.5	10.9	332.9	7.4	714.8	13.1	370,9	8,3	1,3	0,0	99,6	2,3
Wheat	19.1	3.3	25.0	3.0	17.0	3.0	36.5	6.0	18,9	3,1	0,1	0,0	5,1	1,0
Barley	40.9	7.8	53.7	7.7	36.5	6.9	78.4	16.8	40,7	6,9	0,1	0,0	10,9	2,4
Oats	37.0	9.5	48.5	9.5	33.0	8.5	70.8	21.4	36,7	8,3	0,1	0,0	9,9	3,2
Soya	134.9	47.4	177.0	60.5	120.3	42.2	258.4	74.0	134,1	49,5	0,5	0,2	36,0	12,9
Rapeseed	73.4	23.4	96.2	21.4	65.4	22.0	140.5	46.6	72,9	21,3	0,2	0,1	19,6	6,9
Peas	42.1	18.2	55.2	15.4	37.5	16.2	80.6	23.2	41,8	13,7	0,1	0,1	11,2	4,5
Broad beans	42.1	17.4	55.2	15.4	37.5	15.5	80.6	32.4	41,8	16,3	0,1	0,1	11,2	6,5
Linseed	0.6	0.5	0.8	0.7	0.6	0.4	1.2	1.0	0,6	0,5	0,0	0,0	0,2	0,1
Brewer's yeast	16.9	9.7	22.2	12.7	15.1	8.7	32.4	19.3	16,8	10,0	0,1	0,0	4,5	3,0
Sugar beet	73.7	1.1	96.6	1.1	65.7	1.0	141.1	2.1	73,2	0,8	0,3	0,0	19,7	0,3
Potato	8.9	0.3	11.6	0.4	7.9	0.2	17.0	0.7	8,8	0,2	0,0	0,0	2,4	0,1
Sweet lupine	0.7	0.4	0.9	0.4	0.6	0.3	1.3	0.7	0,7	0,3	0,0	0,0	0,2	0,1
Sum of area (ha)	303286.3		320611.1		313349.1		821736.3		304354.5		1145.6		75112.6	

	AT	DE	CH	IT	FR	LI	SI
**Slaughter animals (n)**							
Bull/stores	2048.4	2551.9	1652.5	3387.3	1681.1	5.5	415.3
Calves	99820.0	130901.9	89006.0	191130.8	99168.3	339.1	26629.7
Milk cow	248454.4	325817.8	221538.0	475729.0	246832.3	844.1	66282.1
Breeding cattle, young bulls	406130.5	532591.0	362132.4	777640.1	403479.0	1379.7	108346.5
**Slaughter age (a)**							
Bull/Stores	19.0	20.0	21.0	22.0	23.0	24.0	25.0
Calves	0.3	0.3	0.3	0.3	0.3	0.3	0.3
Milk cow	4.5	4.5	4.5	4.5	4.5	4.5	4.5
Breeding cattle, young bulls	1.2	1.2	1.2	1.2	1.2	1.2	1.2
**Live weight (kg)**							
Bull/Stores	698.8	698.8	698.8	698.8	698.8	698.8	698.8
Calves	169.6	169.6	169.6	169.6	169.6	169.6	169.6
Milk cow	666.9	666.9	666.9	666.9	666.9	666.9	666.9
Breeding cattle, young bulls	315.0	315.0	315.0	315.0	315.0	315.0	315.0
**Triggered slaughter weight (%, boneless)**							
Bull/Stores	41.3	41.3	41.3	41.3	41.3	41.3	41.3
Calves	37.5	37.5	37.5	37.5	37.5	37.5	37.5
Milk cow	37.5	37.5	37.5	37.5	37.5	37.5	37.5
Breeding cattle, young bulls	45.0	45.0	45.0	45.0	45.0	45.0	45.0
**Triggered slaughter weight for the whole herd (t)**							
Bull/Stores	590.4	735.5	476.3	976.3	484.5	1.6	119.7
Calves	19043.4	24973.1	16980.3	36463.5	18919.1	64.7	5080.4
Milk cow	62137.2	81485.4	55405.6	118977.4	61731.5	211.1	16576.8
Breeding cattle, young bulls	57569.0	75494.8	51332.3	110230.5	57193.2	195.6	15358.1
Sum of net meat weight	139340.0	182688.9	124194.5	266647.7	138328.3	473.0	37135.0
**Meat production (t ha^{-1})**	0,0897	0,1492	0,1212	0,1181	0,1353	0,1061	0,1233

Appendix 9: Current horsemeat production: feed demand and required production area per crop and country. The data are based on the data in Appendices 3 (areal yield), 4 (feed composition) and 5 (energy demand), as well as the data on cattle per country (cf. data source for livestock in Appendix 6). The calculation of meat yield is based on slaughter information (source: https://publikationen.sachsen.de).

	**AT**		**DE**		**CH**		**IT**		**FR**		**LI**		**SI**	
	Demand (10^3 t a^{-1})	Area (10^3 ha)	Demand (10^3 t a^{-1})	Area (10^3 ha)	Demand (10^3 t a^{-1})	Area (10^3 ha)	Demand (10^3 t a^{-1})	Area (10^3 ha)	Demand (10^3 t a^{-1})	Area (10^3 ha)	Demand (10^3 t a^{-1})	Area (10^3 ha)	Demand (10^3 t a^{-1})	Area (10^3 ha)
Grass/ hay	98.7	17.3	278.6	38.1	548.9	130.8	427.2	153,4	1529,4	294,0	4,6	1,0	190,3	25,2
Straw	35.9	1.2	101.3	3.4	199.6	6.7	155.4	5,2	556,1	18,6	1,7	0,1	69,2	2,3
Maize	9.6	0.9	27.0	2.5	53.3	5.3	41.5	3,8	148,5	16,2	0,4	0,0	18,5	2,3
Wheat	3.6	0.6	10.1	1.2	20.0	3.5	15.6	2,5	55,7	9,1	0,2	0,0	6,9	1,4
Barley	4.8	0.9	13.5	1.9	26.6	5.1	20.7	4,5	74,2	12,6	0,2	0,0	9,2	2,0
Oats	41.3	10.7	116.6	23.0	229.8	59.4	178.8	54,1	640,2	144,0	1,9	0,5	79,7	25,9
Soya	3.6	1.3	10.1	3.5	20.0	7.0	15.6	4,5	55,7	20,5	0,2	0,1	6,9	2,5
Linseed	2.4	1.9	6.8	5.4	13.3	10.6	10.4	8,3	37,1	29,5	0,1	0,1	4,6	3,7
Carrots	3.1	0.1	8.8	0.1	17.4	0.3	13.5	0,3	48,5	1,1	0,1	0,0	6,0	0,3
Brewer's yeast	1.2	0.7	3.5	2.0	6.8	3.9	5.3	3,2	19,0	11,3	0,1	0,0	2,4	1,6
Sugar beet	2.4	0.0	6.8	0.1	13.5	0.2	10.5	0,2	37,5	0,4	0,1	0,0	4,7	0,1
Sum of area (ha)	35680.1		81218.5		232763.9		239916.8		557386.4		1850.5		67170.6	
**Slaughter animals (n)**														
Mare	4237.7		11957.0		23556.6		18334.9		65635.6		195.7		8166.5	
Young horse	0.0		0.0		0.0		0.0		0.0		0.0		0.0	
Foal	2833.2		7994.1		15749.2		12258.2		43882.1		130.8		5459.9	
Stallion	1430.2		4035.5		7950.3		6188.0		22152.0		66.0		2756.2	
**Slaughter age (a)**														
Mare	9.0		9.0		9.0		9.0		9.0		9.0		9.0	
Young horse	2.0		2.0		2.0		2.0		2.0		2.0		2.0	
Foal	0.5		0.5		0.5		0.5		0.5		0.5		0.5	
Stallion	8.0		8.0		8.0		8.0		8.0		8.0		8.0	
**Live weight (kg)**														
Mare	750.0		750.0		750.0		750.0		750.0		750.0		750.0	
Young horse	750.0		750.0		750.0		750.0		750.0		750.0		750.0	
Foal	280.0		280.0		280.0		280.0		280.0		280.0		280.0	
Stallion	800.0		800.0		800.0		800.0		800.0		800.0		800.0	
**Triggered slaughter weight (%, boneless)**														
Mare	48.8		48.8		48.8		48.8		48.8		48.8		48.8	
Young horse	48.8		48.8		48.8		48.8		48.8		48.8		48.8	
Foal	52.5		52.5		52.5		52.5		52.5		52.5		52.5	
Stallion	48.8		48.8		48.8		48.8		48.8		48.8		48.8	
**Triggered slaughter weight for the whole herd (t)**														
Mare	1549.4		4371.8		8612.9		6703.7		23998.0		71.5		2985.9	
Young horse	0.0		0.0		0.0		0.0		0.0		0.0		0.0	
Foal	416.5		1175.1		2315.1		1802.0		6450.7		19.2		802.6	
Stallion	557.8		1573.8		3100.6		2413.3		8639.3		25.8		1074.9	
Sum of net meat weight	2523.7		7120.7		14028.6		10919.0		39088.0		116.5		4863.4	
**Meat production (t ha^{-1})**	0.0707		0.0877		0.0603		0.0455		0.0701		0.0630		0.0724	

Appendix 10: Current sheep meat production: feed demand and required production area per crop and country. The data are based on the data in Appendices 3 (areal yield), 4 (feed composition) and 5 (energy demand), as well as the data on cattle per country (cf. data source for livestock in Appendix 6). The calculation of meat yield is based on slaughter information (source: www.ama.at/).

	**AT**		**DE**		**CH**		**IT**		**FR**		**LI**		**SI**	
	Demand (10^3 t a^{-1})	Area (10^3 ha)	Demand (10^3 t a^{-1})	Area (10^3 ha)	Demand (10^3 t a^{-1})	Area (10^3 ha)	Demand (10^3 t a^{-1})	Area (10^3 ha)	Demand (10^3 t a^{-1})	Area (10^3 ha)	Demand (10^3 t a^{-1})	Area (10^3 ha)	Demand (10^3 t a^{-1})	Area (10^3 ha)
Grass/ hay	167.3	29.4	25.7	3.5	39.4	9.4	66.8	24,0	81,6	15,7	0,1	0,0	13,5	1,8
Maize	22.1	2.2	3.4	0.3	5.2	0.5	8.8	0,8	10,8	1,2	0,0	0,0	1,8	0,2
Wheat	20.3	3.5	3.1	0.4	4.8	0.8	8.1	1,3	9,9	1,6	0,0	0,0	1,6	0,3
Barley	29.7	5.6	4.6	0.7	7.0	1.3	11.8	2,5	14,5	2,5	0,0	0,0	2,4	0,5
Oats	8.5	2.2	1.3	0.3	2.0	0.5	3.4	1,0	4,1	0,9	0,0	0,0	0,7	0,2
Soya	1.0	0.3	0.1	0.1	0.2	0.1	0.4	0,1	0,5	0,2	0,0	0,0	0,1	0,0
Broad beans	22.9	9.5	3.5	1.0	5.4	2.2	9.1	3,7	11,2	4,4	0,0	0,0	1,9	1,1
Sum of area (ha)	52748.5		6146.6		14913.2		33495.6		26398.4		44.2		4183.9	

	AT	DE	CH	IT	FR	LI	SI
**Slaughter animals (n)**							
Ewe	43869.6	5610.3	7389.9	10953.3	11885.2	16.1	1612.9
Young sheep	45331.9	6956.7	10690.7	18109.5	22106.4	33.4	3666.6
Fattening lamb	59224.0	9088.6	13966.8	23659.1	28881.0	43.6	4790.3
Ram	3655.8	561.0	862.2	1460.4	1782.8	2.7	295.7
**Slaughter age (a)**							
Ewe	5.0	5.0	5.0	5.0	5.0	5.0	5.0
Young sheep	1.0	1.0	1.0	1.0	1.0	1.0	1.0
Fattening lamb	0.5	0.5	0.5	0.5	0.5	0.5	0.5
Ram	5	5	5	5	5	5	5
**Live weight (kg)**							
Ewe	73.0	73.0	73.0	73.0	73.0	73.0	73.0
Young sheep	60.0	60.0	60.0	60.0	60.0	60.0	60.0
Fattening lamb	44.0	44.0	44.0	44.0	44.0	44.0	44.0
Ram	110.0	110.0	110.0	110.0	110.0	110.0	110.0
**Triggered slaughter weight (%, boneless)**							
Ewe	41.3	41.3	41.3	41.3	41.3	41.3	41.3
Young sheep	41.3	41.3	41.3	41.3	41.3	41.3	41.3
Fattening lamb	41.3	41.3	41.3	41.3	41.3	41.3	41.3
Ram	41.3	41.3	41.3	41.3	41.3	41.3	41.3
**Triggered slaughter weight for the whole herd (t)**							
Ewe	1321.0	202.7	311.5	527.7	644.2	1.0	106.8
Young sheep	1122.0	172.2	264.6	448.2	547.1	0.8	90.7
Fattening lamb	1074.9	165.0	253.5	429.4	524.2	0.8	86.9
Ram	165.9	25.5	39.1	66.3	80.9	0.1	13.4
Sum of net meat weight	3683.8	565.3	868.7	1471.6	1796.4	2.7	298.0
**Meat production (t ha^{-1})**	0.0698	0.0920	0.0583	0.0439	0.0681	0.0614	0.0712

Appendix 11: Current goat meat production: feed demand and required production area per crop and country. The data are based on the data in Appendices 3 (areal yield), 4 (feed composition) and 5 (energy demand), as well as the data on cattle per country (cf. data source for livestock in Appendix 6). The calculation of meat yield is based on slaughter information (source: www.ama.at/).

	**AT**		**DE**		**CH**		**IT**		**FR**		**LI**		**SI**	
	Demand (10^3 t a^{-1})	Area (10^3 ha)	Demand (10^3 t a^{-1})	Area (10^3 ha)	Demand (10^3 t a^{-1})	Area (10^3 ha)	Demand (10^3 t a^{-1})	Area (10^3 ha)	Demand (10^3 t a^{-1})	Area (10^3 ha)	Demand (10^3 t a^{-1})	Area (10^3 ha)	Demand (10^3 t a^{-1})	Area (10^3 ha)
Grass/ hay	35.5	6.2	859.5	117.6	722.3	172.2	3152.4	1131,8	261,0	50,2	0,8	0,2	151,6	20,1
Maize	4.7	0.5	113.5	10.5	95.4	9.4	416.2	38,5	34,5	3,8	0,1	0,0	20,0	2,5
Wheat	4.3	0.8	104.5	12.7	87.8	15.3	383.3	62,8	31,7	5,2	0,1	0,0	18,4	3,7
Barley	6.3	1.2	152.4	21.9	128.1	24.3	559.0	120,0	46,3	7,9	0,1	0,0	26,9	5,9
Oats	1.8	0.5	43.5	8.6	36.6	9.5	159.7	48,4	13,2	3,0	0,0	0,0	7,7	2,5
Soya	0.2	0.1	4.9	1.7	4.2	1.5	18.1	5,2	1,5	0,6	0,0	0,0	0,9	0,3
Broad beans	4.9	2.0	117.6	32.8	98.8	40.9	431.3	173,6	35,7	14,0	0,1	0,0	20,7	12,0

	**AT**	**DE**	**CH**	**IT**	**FR**	**LI**	**SI**
Sum of area (ha)	10616.7	194950.2	258650.4	1496875.4	79999.0	257.3	44423.0
**Slaughter animals (n)**							
Milk goat	9322.1	225435.9	189442.4	826861.3	68447.0	198.6	39775.8
Young goat	16344.7	395264.3	332155.7	1449763.5	120010.5	348.2	69740.3
Goat lamb	15940.8	385495.4	323946.6	1413932.9	117044.4	339.6	68016.6
Billy goat	647.4	15655.3	13155.7	57420.9	4753.3	13.8	2762.2
**Slaughter age (a)**							
Milk goat	5.0	5.0	5.0	5.0	5.0	5.0	5.0
Young goat	0.3	0.3	0.3	0.3	0.3	0.3	0.3
Goat lamb	0.2	0.2	0.2	0.2	0.2	0.2	0.2
Billy goat	6.0	6.0	6.0	6.0	6.0	6.0	6.0
**Live weight (kg)**							
Milk goat	60.5	60.5	60.5	60.5	60.5	60.5	60.5
Young goat	35.0	35.0	35.0	35.0	35.0	35.0	35.0
Goat lamb	16.5	16.5	16.5	16.5	16.5	16.5	16.5
Billy goat	80.0	80.0	80.0	80.0	80.0	80.0	80.0
**Triggered slaughter weight (%, boneless)**							
Milk goat	41.3	41.3	41.3	41.3	41.3	41.3	41.3
Young goat	41.3	41.3	41.3	41.3	41.3	41.3	41.3
Goat lamb	41.3	41.3	41.3	41.3	41.3	41.3	41.3
Billy goat	41.3	41.3	41.3	41.3	41.3	41.3	41.3
**Triggered slaughter weight for the whole herd (t)**							
Milk goat	232.6	5626.0	4727.8	20635.4	1708.2	5.0	992.7
Young goat	236.0	5706.6	4795.5	20931.0	1732.7	5.0	1006.9
Goat lamb	108.5	2623.8	2204.9	9623.6	796.6	2.3	462.9
Billy goat	21.4	516.6	434.1	1894.9	156.9	0.5	91.2
Sum of net meat weight	598.5	14473.1	12162.3	53084.8	4394.3	12.8	2553.6
**Meat production (t ha^{-1})**	0.0564	0.0742	0.0470	0.0355	0.0549	0.0496	0.0575

Appendix 12: Current pork production: feed demand and required production area per crop and country.
The data are based on the data in Appendices 3 (areal yield), 4 (feed composition) and 5 (energy demand), as well as the data on cattle per country (cf. data source for livestock in Appendix 6). The calculation of meat yield is based on slaughter information (source: www.ama.at/).

	AT		DE		CH		IT		FR		LI		SI	
	Demand (10^3 t a^{-1})	Area (10^3 ha)	Demand (10^3 t a^{-1})	Area (10^3 ha)	Demand (10^3 t a^{-1})	Area (10^3 ha)	Demand (10^3 t a^{-1})	Area (10^3 ha)	Demand (10^3 t a^{-1})	Area (10^3 ha)	Demand (10^3 t a^{-1})	Area (10^3 ha)	Demand (10^3 t a^{-1})	Area (10^3 ha)
Grass/ hay	10.1	1.8	17.1	2.3	44.7	10.7	326.3	117,2	65,2	12,5	0,0	0,0	24,1	3,2
Alfalfa	16.4	1.1	27.7	1.6	72.3	4.1	527.6	23,0	105,4	5,8	0,1	0,0	39,0	2,2
Maize	172.4	17.1	291.4	27.0	761.8	75.4	5557.8	514,6	1110,8	121,2	0,7	0,1	411,1	50,3
Wheat	169.9	29.5	287.2	34.8	750.8	130.6	5477.8	897,5	1094,8	179,4	0,7	0,1	405,1	80,8
Barley	141.9	27.0	239.8	34.5	627.0	119.2	4574.6	982,3	914,3	155,1	0,6	0,1	338,3	74,6
Triticale	96.2	18.1	162.7	22.6	425.4	79.9	3103.4	563,0	620,2	112,5	0,4	0,1	229,5	52,3
Oats	34.4	8.9	58.2	11.5	152.2	39.3	1110.2	336,2	221,9	49,9	0,1	0,0	82,1	26,7
Soya	76.5	26.8	129.3	44.2	338.1	118.6	2467.0	706,7	493,0	182,0	0,3	0,1	182,5	65,4
Rapeseed	47.9	15.3	81.0	18.0	211.8	71.1	1545.6	512,9	308,9	90,3	0,2	0,1	114,3	40,0
Sunflowers	21.5	8.3	36.4	10.6	95.1	36.9	693.7	194,3	138,6	56,2	0,1	0,0	51,3	22,2
Lucerne meal	10.3	1.5	17.3	2.5	45.3	6.6	330.9	47,9	66,1	9,6	0,0	0,0	24,5	3,5
Peas	35.2	15.2	59.5	16.6	155.7	67.3	1135.6	327,6	227,0	74,2	0,2	0,1	84,0	33,8
Broad beans	27.4	11.4	46.4	13.0	121.3	50.3	885.1	356,3	176,9	69,2	0,1	0,0	65,5	37,7
Linseed	35.5	28.2	60.0	47.7	156.8	124.8	1143.8	910,3	228,6	181,9	0,2	0,1	84,6	67,3
Brewer's yeast	42.9	24.7	72.6	41.7	189.8	109.0	1384.5	824,8	276,7	164,8	0,2	0,1	102,4	68,6
Sugar beet	120.1	1.7	203.0	2.2	530.8	7.8	3872.6	57,3	774,0	8,5	0,5	0,0	286,4	3,8
Potato	148.4	4.6	250.8	9.1	655.8	20.2	4784.4	200,6	956,2	23,0	0,6	0,0	353,9	14,2
Manioc	46.4	5.9	78.4	9.9	205.0	25.9	1495.4	188,7	298,9	37,7	0,2	0,0	110,6	14,0
Rye	139.4	32.6	235.7	41.4	616.3	144.3	4496.2	1224,8	898,6	207,9	0,6	0,1	332,5	88,8
Sweet lupine	24.1	12.3	40.8	19.9	106.6	54.5	778.0	379,3	155,5	69,4	0,1	0,1	57,5	28,1
Milk products (13% DM)	56.1	70.1	94.8	60.2	247.8	257.6	1808.2	1227.3	361,4	269.4	0,2	0,3	133,7	121.0
Fodder oil	5.2	6.1	8.7	7.7	22.8	27.7	166.7	187,3	33,3	46,7	0,0	0,0	12,3	12,5
Sum of area (ha)	307237.0		426659.2		1357404.2		9711818.9		1892988.1		1319.3		805609.9	
**Slaughter animals (n)**														
Porker	4874310.5		8239728.3		21542425.3		157172383.4		31411776.0		21033.3		11624490.0	
Breeding sow	101198.2		171069.4		447253.8		3263140.1		652156.7		436.7		241342.3	
Breeding boars	809.1		1367.7		3575.7		26088.3		5213.9		3.5		1929.5	
**Slaughter age (a)**														
Porker	0.5		0.5		0.5		0.5		0.5		0.5		0.5	
Breeding sow	3.6		3.6		3.6		3.6		3.6		3.6		3.6	
Breeding boars	10.0		10.0		10.0		10.0		10.0		10.0		10.0	
**Live weight (kg)**														
Porker	118.9		118.9		118.9		118.9		118.9		118.9		118.9	
Breeding sow	236.3		236.3		236.3		236.3		236.3		236.3		236.3	
Breeding boars	236.3		236.3		236.3		236.3		236.3		236.3		236.3	
**Triggered slaughter weight (%, boneless)**														
Porker	80.6		80.6		80.6		80.6		80.6		80.6		80.6	
Breeding sow	75.4		75.4		75.4		75.4		75.4		75.4		75.4	
Breeding boars	75.4		75.4		75.4		75.4		75.4		75.4		75.4	
**Triggered slaughter weight for the whole herd (t)**														
Porker	467115.9		789631.4		2064458.2		15062176.5		3010259.8		2015.7		1114000.5	
Breeding sow	18017.6		30457.7		79630.4		580979.5		116112.0		77.7		42969.3	
Breeding boars	144.0		243.5		636.6		4644.8		928.3		0.6		343.5	
Sum of net meat weight	485277.6		820332.6		2144725.3		15647800.8		3127300.1		2094.0		1157313.4	
**Meat production (t ha^{-1})**	1,3180		1,7124		1,3559		1,4516		1,4700		1,3608		1,2706	

Appendix 13: Current chicken meat production: feed demand and required production area per crop and country. Area calculations are based on the number of flocks excluding laying hens, as these are already included in egg production (cf. Appendix 7).
The data are based on the data in Appendices 3 (areal yield), 4 (feed composition) and 5 (energy demand), as well as the data on cattle per country (cf. data source for livestock in Appendix 6). The calculation of meat yield is based on slaughter information (source: www.ama.at/).

	AT		DE		CH		IT		FR		LI		SI	
	Demand (10^3 t a^{-1})	Area (10^3 ha)	Demand (10^3 t a^{-1})	Area (10^3 ha)	Demand (10^3 t a^{-1})	Area (10^3 ha)	Demand (10^3 t a^{-1})	Area (10^3 ha)	Demand (10^3 t a^{-1})	Area (10^3 ha)	Demand (10^3 t a^{-1})	Area (10^3 ha)	Demand (10^3 t a^{-1})	Area (10^3 ha)
Alfalfa	0.1	0.0	1.0	0.1	0.1	0.0	0.5	0,0	1,2	0,1	0,0	0,0	0,1	0,0
Maize	28.8	2.8	235.4	21.8	29.2	2.9	127.4	11,8	308,0	33,6	0,0	0,0	15,8	1,9
Wheat	48.0	8.3	392.1	47.5	48.7	8.5	212.3	34,8	512,9	84,0	0,0	0,0	26,3	5,2
Barley	3.3	0.6	26.6	3.8	3.3	0.6	14.4	3,1	34,8	5,9	0,0	0,0	1,8	0,4
Triticale	4.6	0.9	37.3	5.2	4.6	0.9	20.2	3,7	48,8	8,9	0,0	0,0	2,5	0,6
Oats	0.1	0.0	1.2	0.2	0.1	0.0	0.6	0,2	1,5	0,3	0,0	0,0	0,1	0,0
Millet	0.2	0.1	1.9	0.6	0.2	0.1	1.0	0,3	2,5	0,8	0,0	0,0	0,1	0,1
Sesame	0.2	0.2	1.5	1.3	0.2	0.2	0.8	0,7	2,0	1,7	0,0	0,0	0,1	0,1
Soya	25.3	8.9	207.2	70.9	25.7	9.0	112.1	32,1	271,0	100,0	0,0	0,0	13,9	5,0
Rapeseed	5.8	1.8	47.1	10.5	5.8	2.0	25.5	8,5	61,5	18,0	0,0	0,0	3,2	1,1
Sunflowers	6.0	2.3	48.9	14.3	6.1	2.4	26.5	7,4	64,0	25,9	0,0	0,0	3,3	1,4
Lucerne meal	4.0	0.6	32.8	4.7	4.1	0.6	17.7	2,6	42,9	6,2	0,0	0,0	2,2	0,3
Peas	3.5	1.5	28.6	8.0	3.6	1.5	15.5	4,5	37,4	12,2	0,0	0,0	1,9	0,8
Broad beans	0.5	0.2	3.7	1.0	0.5	0.2	2.0	0,8	4,8	1,9	0,0	0,0	0,2	0,1
Linseed	0.4	0.3	3.5	2.8	0.4	0.3	1.9	1,5	4,6	3,7	0,0	0,0	0,2	0,2
Fodder oil	4.8	5.7	39.4	34.8	4.9	5.9	21.4	24,0	51,6	72,3	0,0	0,0	2,6	2,7
Sum of area (ha)	34341.1		227496.6		35088.0		135919.6		375585.9		33.9		19928.1	
**Slaughter animals (n)**														
Chicken for fattening	56389014.0		461054741.4		57253525.3		249572731.5		603080906.5		55900.5		30894526.5	
Laying hen	3559599.5		29104432.4		3614172.5		15754469.1		38069942.5		3528.8		1950240.6	
Parent animal	37868.1		309621.6		38448.6		167600.7		404999.4		37.5		20747.2	
**Slaughter age (a)**														
Chicken for fattening	0.1		0.1		0.1		0.1		0.1		0.1		0.1	
Laying hen	1.5		1.5		1.5		1.5		1.5		1.5		1.5	
Parent animal	1.5		1.5		1.5		1.5		1.5		1.5		1.5	
**Live weight (kg)**														
Chicken for fattening	1.5		1.5		1.5		1.5		1.5		1.5		1.5	
Laying hen	1.8		1.8		1.8		1.8		1.8		1.8		1.8	
Parent animal	1.8		1.8		1.8		1.8		1.8		1.8		1.8	
**Triggered slaughter weight (%, boneless)**														
Chicken for fattening	66.0		66.0		66.0		66.0		66.0		66.0		66.0	
Laying hen	66.0		66.0		66.0		66.0		66.0		66.0		66.0	
Parent animal	66.0		66.0		66.0		66.0		66.0		66.0		66.0	
**Triggered slaughter weight for the whole herd (t)**														
Chicken for fattening	55825.1		456444.2		56681.0		247077.0		597050.1		55.3		30585.6	
Laying hen	4111.3		33615.6		4174.4		18196.4		43970.8		4.1		2252.5	
Parent animal	43.7		357.6		44.4		193.6		467.8		0.0		24.0	
Sum of net meat weight	59980.2		490417.4		60899.8		265467.0		641488.7		59.5		32862.1	
**Meat production (t ha^{-1})**	0.7653		0.9446		0.7605		0.8558		0.7484		0.7690		0.7226	

Appendix 14: Current goose meat production: feed demand and required production area per crop and country. The data are based on the data in Appendices 3 (areal yield), 4 (feed composition) and 5 (energy demand), as well as the data on cattle per country (cf. data source for livestock in Appendix 6). The calculation of meat yield is based on slaughter information (source: https://publikationen.sachsen.de).

	**AT**		**DE**		**CH**		**IT**		**FR**		**LI**		**SI**	
	Demand (10^3 t a^{-1})	Area (10^3 ha)	Demand (10^3 t a^{-1})	Area (10^3 ha)	Demand (10^3 t a^{-1})	Area (10^3 ha)	Demand (10^3 t a^{-1})	Area (10^3 ha)	Demand (10^3 t a^{-1})	Area (10^3 ha)	Demand (10^3 t a^{-1})	Area (10^3 ha)	Demand (10^3 t a^{-1})	Area (10^3 ha)
Alfalfa	0.0	0.0	0.0	0.0	0.0	0.0	0.0	0,0	0,0	0,0	0,0	0,0	0,0	0,0
Maize	0.2	0.0	0.2	0.0	0.0	0.0	5.9	0,5	2,7	0,3	0,0	0,0	0,1	0,0
Wheat	0.3	0.1	0.4	0.0	0.0	0.0	9.8	1,6	4,5	0,7	0,0	0,0	0,2	0,0
Barley	0.0	0.0	0.0	0.0	0.0	0.0	0.7	0,1	0,3	0,1	0,0	0,0	0,0	0,0
Triticale	0.0	0.0	0.0	0.0	0.0	0.0	0.9	0,2	0,4	0,1	0,0	0,0	0,0	0,0
Oats	0.0	0.0	0.0	0.0	0.0	0.0	0.0	0,0	0,0	0,0	0,0	0,0	0,0	0,0
Millet	0.0	0.0	0.0	0.0	0.0	0.0	0.0	0,0	0,0	0,0	0,0	0,0	0,0	0,0
Sesame	0.0	0.0	0.0	0.0	0.0	0.0	0.0	0,0	0,0	0,0	0,0	0,0	0,0	0,0
Soya	0.2	0.1	0.2	0.1	0.0	0.0	5.2	1,5	2,4	0,9	0,0	0,0	0,1	0,0
Rapeseed	0.0	0.0	0.0	0.0	0.0	0.0	1.2	0,4	0,5	0,2	0,0	0,0	0,0	0,0
Sunflowers	0.0	0.0	0.0	0.0	0.0	0.0	1.2	0,3	0,6	0,2	0,0	0,0	0,0	0,0
Lucerne meal	0.0	0.0	0.0	0.0	0.0	0.0	0.8	0,1	0,4	0,1	0,0	0,0	0,0	0,0
Peas	0.0	0.0	0.0	0.0	0.0	0.0	0.7	0,2	0,3	0,1	0,0	0,0	0,0	0,0
Broad beans	0.0	0.0	0.0	0.0	0.0	0.0	0.1	0,0	0,0	0,0	0,0	0,0	0,0	0,0
Linseed	0.0	0.0	0.0	0.0	0.0	0.0	0.1	0,1	0,0	0,0	0,0	0,0	0,0	0,0
Fodder oil	0.0	0.0	0.0	0.0	0.0	0.0	1.0	1,1	0,5	0,6	0,0	0,0	0,0	0,0
Sum of area (ha)	212.1		217.8		1.4		6290.0		3284.4		0.0		115.3	
**Slaughter animals (n)**														
Fattening goose	91651.3		116163.7		619.5		3039245.3		1387800.0		0.0		47042.1	
Parent animal	169.7		215.1		1.1		5628.2		2570.0		0.0		87.1	
**Slaughter age (a)**														
Fattening goose	0.3		0.3		0.3		0.3		0.3		0.3		0.3	
Parent animal	20.0		20.0		20.0		20.0		20.0		20.0		20.0	
**Live weight (kg)**														
Fattening goose	5.0		5.0		5.0		5.0		5.0		5.0		5.0	
Parent animal	8.0		8.0		8.0		8.0		8.0		8.0		8.0	
**Triggered slaughter weight (%, boneless)**														
Fattening goose	55.0		55.0		55.0		55.0		55.0		55.0		55.0	
Parent animal	50.0		50.0		50.0		50.0		50.0		50.0		50.0	
**Triggered slaughter weight for the whole herd (t)**														
Fattening goose	252.0		319.5		1.7		8357.9		3816.5		0.0		129.4	
Parent animal	0.7		0.9		0.0		22.5		10.3		0.0		0.3	
Sum of net meat weight	252.7		320.3		1.7		8380.4		3826.7		0.0		129.7	
**Meat production (t ha^{-1})**	1.1915		1.4705		1.1840		1.3323		1.1651				1.1249	

Appendix 15: Current duck and guinea fowl meat production: feed demand and required production area per crop and country. The data are based on the data in Appendices 3 (areal yield), 4 (feed composition) and 5 (energy demand), as well as the data on cattle per country (cf. data source for livestock in Appendix 6). The calculation of meat yield is based on slaughter information (source: https://publikationen.sachsen.de).

	AT		DE		CH		IT		FR		LI		SI	
	Demand (10^3 t a^{-1})	Area (10^3 ha)	Demand (10^3 t a^{-1})	Area (10^3 ha)	Demand (10^3 t a^{-1})	Area (10^3 ha)	Demand (10^3 t a^{-1})	Area (10^3 ha)	Demand (10^3 t a^{-1})	Area (10^3 ha)	Demand (10^3 t a^{-1})	Area (10^3 ha)	Demand (10^3 t a^{-1})	Area (10^3 ha)
Alfalfa	0.0	0.0	0.1	0.0	0.0	0.0	0.0	0,0	1,0	0,1	0,0	0,0	0,0	0,0
Maize	0.0	0.0	17.9	1.7	0.5	0.0	6.2	0,6	244,0	26,6	0,0	0,0	0,7	0,1
Wheat	0.0	0.0	29.9	3.6	0.8	0.1	10.4	1,7	406,4	66,6	0,0	0,0	1,2	0,2
Barley	0.0	0.0	2.0	0.3	0.1	0.0	0.7	0,2	27,6	4,7	0,0	0,0	0,1	0,0
Triticale	0.0	0.0	2.8	0.4	0.1	0.0	1.0	0,2	38,7	7,0	0,0	0,0	0,1	0,0
Oats	0.0	0.0	0.1	0.0	0.0	0.0	0.0	0,0	1,2	0,3	0,0	0,0	0,0	0,0
Millet	0.0	0.0	0.1	0.0	0.0	0.0	0.1	0,0	2,0	0,7	0,0	0,0	0,0	0,0
Sesame	0.0	0.0	0.1	0.1	0.0	0.0	0.0	0,0	1,6	1,3	0,0	0,0	0,0	0,0
Soya	0.0	0.0	15.8	5.4	0.4	0.1	5.5	1,6	214,7	79,2	0,0	0,0	0,6	0,2
Rapeseed	0.0	0.0	3.6	0.8	0.1	0.0	1.2	0,4	48,8	14,3	0,0	0,0	0,1	0,0
Sunflowers	0.0	0.0	3.7	1.1	0.1	0.0	1.3	0,4	50,7	20,5	0,0	0,0	0,1	0,1
Lucerne meal	0.0	0.0	2.5	0.4	0.1	0.0	0.9	0,1	34,0	4,9	0,0	0,0	0,1	0,0
Peas	0.0	0.0	2.2	0.6	0.1	0.0	0.8	0,2	29,6	9,7	0,0	0,0	0,1	0,0
Broad beans	0.0	0.0	0.3	0.1	0.0	0.0	0.1	0,0	3,8	1,5	0,0	0,0	0,0	0,0
Linseed	0.0	0.0	0.3	0.2	0.0	0.0	0.1	0,1	3,6	2,9	0,0	0,0	0,0	0,0
Fodder oil	0.0	0.0	3.0	2.7	0.1	0.1	1.0	1,2	40,9	57,3	0,0	0,0	0,1	0,1
Sum of area (ha)	9.0		17327.5		557.2		6635.7		297595.4		0.0		873.5	
**Slaughter animals (n)**														
Fattening duck, fowl		7595.6		12862892.6		333043.6		4462969.5		175031952.8		0.0		496006.6
Parent animal		16.1		38282.4		991.2		13282.6		520928.4		0.0		1476.2
**Slaughter age (a)**														
Fattening duck, fowl		0.2		0.3		0.3		0.3		0.3		0.3		0.3
Parent animal		2.0		2.0		2.0		2.0		2.0		2.0		2.0
**Live weight (kg)**														
Fattening duck, fowl		3.3		2.8		2.8		2.8		2.8		2.8		2.8
Parent animal		4.5		4.5		4.5		4.5		4.5		4.5		4.5
**Triggered slaughter weight (%, boneless)**														
Fattening duck, fowl		55		55		55		55		55		55		55
Parent animal		50		50		50		50		50		50		50
**Triggered slaughter weight for the whole herd (t)**														
Fattening duck, fowl		13.8		23346.1		604.5		8100.3		317683.0		0.0		900.3
Parent animal		0.0		86.1		2.2		29.9		1172.1		0.0		3.3
Sum of net meat weight		13.8		23432.3		606.7		8130.2		318855.1		0.0		903.6
**Meat production (t ha^{-1})**		1.5323		1.3523		1.0888		1.2252		1.0714	-			1.0345

Appendix 16: Current turkey meat production: feed demand and required production area per crop and country. The data are based on the data in Appendices 3 (areal yield), 4 (feed composition) and 5 (energy demand), as well as the data on cattle per country (cf. data source for livestock in Appendix 6). The calculation of meat yield is based on slaughter information (source: https://publikationen.sachsen.de).

	**AT**		**DE**		**CH**		**IT**		**FR**		**LI**		**SI**	
	Demand (10^3 t a^{-1})	Area (10^3 ha)	Demand (10^3 t a^{-1})	Area (10^3 ha)	Demand (10^3 t a^{-1})	Area (10^3 ha)	Demand (10^3 t a^{-1})	Area (10^3 ha)	Demand (10^3 t a^{-1})	Area (10^3 ha)	Demand (10^3 t a^{-1})	Area (10^3 ha)	Demand (10^3 t a^{-1})	Area (10^3 ha)
Alfalfa	0.1	0.0	0.5	0.0	1.2	0.1	9.6	0,4	2,0	0,1	0,0	0,0	0,7	0,0
Maize	18.0	1.8	124.5	11.5	301.0	29.8	2369.9	219,4	499,7	54,5	0,3	0,0	165,3	20,2
Wheat	30.0	5.2	207.3	25.1	501.2	87.2	3947.0	646,7	832,2	136,4	0,5	0,1	275,3	54,9
Barley	2.0	0.4	14.1	2.0	34.0	6.5	268.0	57,6	56,5	9,6	0,0	0,0	18,7	4,1
Triticale	2.9	0.5	19.7	2.7	47.7	9.0	375.6	68,1	79,2	14,4	0,0	0,0	26,2	6,0
Oats	0.1	0.0	0.6	0.1	1.5	0.4	11.7	3,5	2,5	0,6	0,0	0,0	0,8	0,3
Millet	0.1	0.0	1.0	0.3	2.5	0.8	19.4	6,4	4,1	1,4	0,0	0,0	1,4	1,0
Sesame	0.1	0.1	0.8	0.7	2.0	1.6	15.5	12,9	3,3	2,7	0,0	0,0	1,1	0,9
Soya	15.9	5.6	109.5	37.5	264.8	92.9	2085.1	597,3	439,6	162,3	0,3	0,1	145,4	52,1
Rapeseed	3.6	1.1	24.9	5.5	60.1	20.2	473.6	157,2	99,9	29,2	0,1	0,0	33,0	11,6
Sunflowers	3.7	1.5	25.9	7.5	62.5	24.2	492.1	137,8	103,8	42,1	0,1	0,0	34,3	14,8
Lucerne meal	2.5	0.4	17.3	2.5	41.9	6.1	329.9	47,7	69,6	10,1	0,0	0,0	23,0	3,3
Peas	2.2	0.9	15.1	4.2	36.6	15.8	287.8	83,0	60,7	19,8	0,0	0,0	20,1	8,1
Broad beans	0.3	0.1	2.0	0.5	4.7	2.0	37.3	15,0	7,9	3,1	0,0	0,0	2,6	1,5
Linseed	0.3	0.2	1.9	1.5	4.5	3.6	35.4	28,2	7,5	5,9	0,0	0,0	2,5	2,0
Fodder oil	3.0	3.6	20.9	18.4	50.4	61.2	397.0	446,0	83,7	117,3	0,0	0,1	27,7	28,0
Sum of area (ha)		21510.8		120280.1		361177.1		2527373.6		609379.7		345.6		208778.1
**Slaughter animals (n)**														
Fattening cock		796236.1		5495106.2		13285225.6		104613838.9		22057639.0		12855.2		7296331.2
Fattening hen		1632284.1		1816930.3		2389009.9		12919178.9		2074227.5		976.0		464518.0
Parent animal		34877.9		240704.4		581938.3		4582443.8		966200.0		563.1		319604.3
**Slaughter age (a)**														
Fattening cock		0.4		0.4		0.4		0.4		0.4		0.4		0.4
Fattening hen		0.2		1.2		2.2		3.2		4.2		5.2		6.2
Parent animal		2.0		2.0		2.0		2.0		2.0		2.0		2.0
**Live weight (kg)**														
Fattening cock		20.0		20.0		20.0		20.0		20.0		20.0		20.0
Fattening hen		10.0		10.0		10.0		10.0		10.0		10.0		10.0
Parent animal		20.0		20.0		20.0		20.0		20.0		20.0		20.0
**Triggered slaughter weight (%, boneless)**														
Fattening cock		60.0		60.0		60.0		60.0		60.0		60.0		60.0
Fattening hen		60.0		60.0		60.0		60.0		60.0		60.0		60.0
Parent animal		60.0		60.0		60.0		60.0		60.0		60.0		60.0
**Triggered slaughter weight for the whole herd (t)**														
Fattening cock		9554.8		65941.3		159422.7		1255366.1		264691.7		154.3		87556.0
Fattening hen		9793.7		67589.8		163408.3		1286750.2		271309.0		158.1		89744.9
Parent animal		418.5		2888.5		6983.3		54989.3		11594.4		6.8		3835.3
Sum of net meat weight		19767.1		136419.5		329814.2		2597105.6		547595.0		319.1		181136.1
**Meat production (t ha^{-1})**		0.9189		1.1342		0.9132		1.0276		0.8986		0.9234		0.8676

Appendix 17: Current production of coarse fish meat (e.g. carp, tench, grassfish): feed demand and required production area per crop and country, as well as average fish production per area (source: [56]). The area data are only valid under the assumption that the fish products can be produced by local freshwater fish farms, although this possibility could be nutritionally problematic.

	**Feed demand per 1 t fish** (t a^{-1})	**AT** Area (10^3 ha)	**DE** Area (10^3 ha)	**CH** Area (10^3 ha)	**IT** Area (10^3 ha)	**FR** Area (10^3 ha)	**LI** Area (10^3 ha)	**SI** Area (10^3 ha)
Pumpkin pie	0.014	0.023	0.023	0.023	0.023	0.023	0.023	0,021
Sunflowers	0.013	0.005	0.004	0.005	0.004	0.005	0.005	0,006
Soya cake	0.005	0.002	0.002	0.002	0.002	0.002	0.002	0,002
Wheat	0.017	0.003	0.003	0.003	0.003	0.003	0.003	0,003
Rape (cake, oil)	0.021	0.007	0.005	0.007	0.007	0.006	0.007	0,007
Wheat	0.321	0.059	0.059	0.059	0.053	0.051	0.059	0,053
Spelt	0.321	0.074	0.057	0.073	0.054	0.051	0.059	0,064
Rye	0.321	0.075	0.075	0.075	0.088	0.081	0.075	0,086
Oats	0.321	0.083	0.063	0.083	0.097	0.072	0.085	0,104
Barley	0.321	0.061	0.046	0.061	0.069	0.055	0.061	0,071
Millet	0.321	0.106	0.106	0.106	0.106	0.106	0.106	0,231
Maize	0.321	0.032	0.030	0.032	0.030	0.035	0.032	0,039
Rice	0.321	0.092	0.092	0.092	0.085	0.084	0.092	0,092
Fish products (meal, oil)	0.130	0.165	0.190	0.168	0.170	0.182	0.172	0,140
Turkey	0.020	0.049	0.030	0.036	0.031	0.035	0.034	0,036
Pig	0.018	0.045	0.038	0.049	0.048	0.044	0.047	0,050
	Sum of area (ha)	0.881	0.823	0.874	0.869	0.834	0.863	1.005
	**Fish production (t ha^{-1})**	1.134	1.215	1.144	1.151	1.200	1.159	0.995

Appendix 18: Current production of predatory fish meat (e.g. trout, char, pike): feed demand and required production area per crop and country, as well as average fish production per area (sources: [57, 56]). The area data are only valid under the assumption that the fish products can be produced by local freshwater fish farms, although this possibility could be nutritionally problematic.

	**Feed demand per 1 t fish** (t a^{-1})	**AT** Area (10^3 ha)	**DE** Area (10^3 ha)	**CH** Area (10^3 ha)	**IT** Area (10^3 ha)	**FR** Area (10^3 ha)	**LI** Area (10^3 ha)	**SI** Area (10^3 ha)
Pumpkin pie	0.174	0.291	0.291	0.291	0.291	0.291	0.291	0,271
Sunflowers	0.164	0.064	0.048	0.064	0.046	0.067	0.064	0,071
Soya cake	0.069	0.024	0.024	0.024	0.020	0.025	0.024	0,025
Wheat	0.211	0.039	0.039	0.039	0.035	0.033	0.039	0,035
Rape (cake, oil)	0.273	0.087	0.061	0.091	0.090	0.080	0.089	0,095
Fish products (meal, oil)	1.660	1.884	2.017	1.900	1.911	1.992	1.924	1,652
Turkey	0.253	0.625	0.384	0.454	0.394	0.445	0.433	0,455
Pig	0.235	0.578	0.486	0.623	0.611	0.559	0.600	0,636
Blood	0.237	0.908	0.673	0.871	0.850	0.828	0.836	0,846
	Sum of area (ha)	4.499	4.023	4.357	4.249	4.319	4.300	4.087
	**Fish production (t ha^{-1})**	0.222	0.249	0.230	0.235	0.232	0.233	0.245

Appendix 19: Formulas used to obtain the sum of the growing degree days (GDD) for each pixel of the study area and validation of the model.

Calculation of the GDD sum per pixel based on the Growing Season Length

First, we determined the start of the growing season (GLS_{Start}) and the end of the growing season (GLS_{End}). To do this, we correlated the GLS layer with elevation data from the Shuttle Radar Topography Mission (SRTM, [58]). The following functions were used for this purpose:

$$GLS_{Start} = 0.039 \cdot a + 20.495$$

$$GLS_{End} = -0.039 \cdot a + 344.510,$$

using the beginning of winter (21 December) as the turning point. We then calculated the GLS as follows:

$$GLS = 365 - GLS_{Start} - (365 - GLS_{End})$$

Using latitude and longitude, we corrected the deviation between our calculated GLS and the official GLS layer (gslETCCDI_Layer.tif, [59, 60]), because our model overestimates the vegetation length in the northeast and underestimates it in the southeast compared to the layer. We performed an interpolation in ArcGIS (ESRI ArcMap, version 10.5.1.i) using focal statistics (r = 2000m).

We then derived the number of vegetation days for each pixel per month (GLS_{Month}) and calculated the GDD sum as follows:

$$GDD = \sum_{k=1}^{12}(GLS_{Month} \cdot (T_{Month} - 1°C),$$

where we used as monthly mean temperature (T_{Month}) the data generated with the ClimateEU v4.63 software package based on the methodology of [61] for the scenario period 1985-2015.

Model validation

We validated our modelled GDD sum by comparing the modelled GDD sum with the current GDD demand. To obtain the current GDD demand, we first calculated the areal yield for each individual crop by linking the agricultural yield per crop to the Used Agricultural Area (UAA) at the NUTS2 level (cf. sources in Appendix 3). We then multiplied the crop yield statistics by the crop-specific GDDs (cf. Appendix 2). For validation, we compared the actual GDD demand with the modelled GDD sum for the four different land-

use types: permanent grassland, arable land, fruit tree and berry plantations, and vineyards. The spatial distribution of the land-use types was obtained from Corine Land Cover (CLC, [62]). Due to the coarse spatial resolution of CLC of 100 m and the large minimum mapping unit of 25 ha for our purposes, we had to correct the area and consequently the modelled GDD sum using statistical areal land use data at the NUTS2 level [63]. For this step, we assumed that the more favourable areas, i.e. those with a higher GDD, are primarily used within the municipality. Thus, we added areas until the statistical area per municipality was reached. The results in Table S19 show that the current GDD demand determined from the regional yield statistics is in good agreement with the area-corrected GDD modelling, except for the Italian Alps. This deviation occurred because the real suitability of the areas is also influenced by sufficient precipitation or artificial irrigation and suitable soil properties. However, these limitations were not taken into account in the GDD modelling, which resulted in a significant overestimation of the modelled GDDs for the Italian Alpine regions, which are characterized by low precipitation [64].

Table S19: Comparison between the current GDD demand, calculated on the basis of production statistics, and the area-corrected, modelled GDDs for four different land use types at the national level in the Alpine Space.

	**Permanent grassland**			**Arable land**			**Fruit tree and berry plantations**			**Vineyards**		
	Σ_{stat} (10^6 GDD)	Σ_{model} (10^6 GDD)	ratio	Σ_{stat} (10^6 GDD)	Σ_{model} (10^6 GDD)	ratio	Σ_{stat} (10^6 GDD)	Σ_{model} (10^6 GDD)	Ratio	Σ_{stat} (10^6 GDD)	Σ_{model} (10^6 GDD)	ratio
Austria	4345.4	5598.3	1.29	2306.6	2499.5	1.08	32.8	34.9	1.06	302.6	279.5	0.92
Germany	3496.0	3411.8	0.98	1966.0	1745.3	0.89	69.8	74.6	1.07	37.4	35.0	0.93
Italy	2209.3	2485.3	1.12	4205.0	6557.4	1.56	311.2	428.0	1.38	590.8	717.9	1.22
Switzerland	1883.3	1884.5	1.00	680.0	671.6	0.99	18.9	19.7	1.04	43.7	37.1	0.85
France	5351.0	5512.6	1.03	1973.5	2229.5	1.13	183.7	219.6	1.20	495.5	591.6	1.19
Liechtenstein	6.9	6.4	0.94	2.2	2.4	1.06	0.01	0.01	1.04	0.03	0.03	1.11
Slovenia	1306.3	1386.7	1.06	438.0	364.6	0.83	32.5	33.9	1.04	53.3	55.7	1.04

Appendix 20: Sum of avoidable food waste from the categories 'retail and other distribution of food', 'restaurants and food services' and 'households' and food consumption for the year 2021 for the single Alpine regions. Sources: Food waste: EU regions: Eurostat, online data code: env_wasfw; CH: https://www.bafu.admin.ch; Consumption: https://www.fao.org/faostat/en/#data/FBS.

	Consumption ($kg\ yr^{-1}\ cap^{-1}$)	Retail and other distribution of food ($kg\ yr^{-1}\ cap^{-1}$)	Restaurants and food services ($kg\ yr^{-1}\ cap^{-1}$)	Households ($kg\ yr^{-1}\ cap^{-1}$)	Total food waste ($kg\ yr^{-1}\ cap^{-1}$)	Total food waste (%)
Austria	697.7	9.4	19.9	82.8	134.1	16.1
Germany	696.1	9.0	22.1	77.0	129.5	15.5
Italy	693.3	7.8	4.3	104.7	136.9	16.9
Switzerland	679.4	31.9	24.0	89.0	318.8	21.3
France	694.1	9.3	15.9	60.0	128.8	12.3
Slovenia	718.9	6.9	18.9	35.7	67.9	8.6

References

1. Pickert, M., Wolter, M., Döring, J. & Borg, H. Hat sich die Zeitspanne von der Saat bis zur Reife für Sommerkulturen in Mitteldeutschland infolge von Temperaturerhöhung verkürzt? *Hercynia N. F.* **48**, 5-20 (2015).
2. Lancashire, P.D., Bleiholder, H., van den Boom, T., Langeluddeke, P., Stauss, R., Weber, E. & Witzenberger, A. A uniform decimal code for growth stages of crops and weeds. *Ann. Appl. Biol.* **119**, 561-601 (1991).
3. Hein, W. & Waschl, H. Wintergetreidesorten für 2018/19. *Landwirt Bio* **5**, 32-35 (2018).
4. Birnbaum, E. Pflanzenbau. Salzwasser-Verlag, Paderborn (2013).
5. Zeise, K., Haller, J., Hartmann, A., Eberl, V., Grieb, M., Haag, J., Schumann, D. & Fritz, M. Sorghum als Biogassubstrat. Präzisierung der Anbauempfehlungen für bayerische Anbaubedingungen. Technologie-und Förderzentrum (TFZ), Straubing (2016).
6. http://www.ricenortheasternus.org/
7. www.gartenjournal.net/saee-und-erntekalender
8. LK Oberösterreich Versuchsergebnisse 2016. LK Oberösterreich, Linz (2016).
9. Prjanischnikow, D.N. Spezieller Pflanzenbau: Der Anbau der Landwirtschaftlichen Kulturpflanzen. Springer, Berlin (1930).
10. pss.uvm.edu/vtcrops/research/PredictingQualityFirstHarvest.html
11. www.krautundrueben.de/waermebedarf-gemuese
12. Medel, G., Medel, F., Huber, A. & McConchie, C. Phenological Development and Growing Degree Days in Gevuina avellana Mol. *Acta horticulturae* **1052**, 355-362 (2014).
13. Razavi, F., Hajilou, J., Tabatabaei, S.J. & Dadpour, M.R. Comparison of chilling and heat requirement in some peach and apricot cultivars. *Research in Plant Biology* **1(2)**, 40-47 (2011).
14. Huglin, P. Biologie et écologie de la vigne. Lavoisier (Edition Tec & Doc), Paris (1986).
15. www.canr.msu.edu/
16. Krüger, E., Josuttis, M., Nestby, R., Toldam-Andersen, T.B., Carlen, C. & Mezzetti, B. Influence of growing conditions at different latitudes of Europe on strawberry growth performance, yield and quality. *Journal of Berry Research* **2**, 143-157 (2012).
17. Stenzel, N.M.C., Neves, C.S.V.J., Marur, C.J., dos Santos Scholz, M.B. & Gomes, J.C. Maturation curves and degree-days accumulation for fruits of 'Folha Murcha' orange trees. *Sci. agric. (Piracicaba, Braz.)* **63/3**, 219-225 (2006).
18. Miller, P., Lanier, W. & Brandt, S. Using growing degree days to predict plant stages. Montana State University-Bozeman, Bozeman (2001).
19. Statistik Austria, Ernteerhebung - Pflanzliche Produktion in Österreich (Feldfrüchte, Gemüse, Obst und Wein). Statistik Austria, Bundesanstalt Statistik Österreich A-1110 Wien (2019).
20. Destatis, Erntemengen & Anbauflächen ausgewählter Anbaukulturen im Zeitvergleich 2016-2018. Statistisches Bundesamt (2019).
21. Statistisches Landesamt Baden-Württemberg, Ernte der Hauptfeldfrüchte in Baden-Württemberg 2017-2018 Statistische Berichte Baden Württemberg. *Agrarwirtschaft Artikel* **3354,** 17001 (2019).
22. Bayerisches Landesamt für Statistik, Bayern Daten 2016, 2017, 2018, 2019 (2019).
23. ISTAT, Coltivazioni e allevamenti 2010 – 2018 Superfice e produzione dati annuali – I-Stat database Istituto nazionale di Statistica (2019).
24. SBV, Agristat, Getreideproduktion, Entwicklung 2014 – 2017 T 07.02.03.01.01 (2018).
25. BFS, Pflanzenproduktion 1985 – 2016 - T 07.02.03.02.01 - SBV – Pflanzenproduktion (2018).
26. Agreste, Statistique Agricole Annuelle Définitive 2016-2017 – Agreste Chiffres et Données 2019 (2019a).
27. Agreste, Données departments grandes cultures - statistique agricole annuelle 2017- 2018 - Moyenne 2013-2017 (2019b).
28. Agreste, Cultures fruitières 2014-2015 – Départements - Agreste - Statistique agricole annuelle (SAA) downloaded 08_2019 (2019c).
29. Agreste, Cultures développées (Pommes de terre et autres tubercules, Légumes frais, melons ou fraises, Fourrages annuels) 2015-2016 – Départements - Agreste - Statistique agricole annuelle (SAA) downloaded 08_2019 (2019d).

30. Amt für Umwelt, Bericht über die wirtschaftliche Entwicklung der Landwirtschaftsbetriebe im Fürstentum Liechtenstein, Agrarbericht 2018. Regierung des Fürstentums Liechtenstein, Vaduz (2020).
31. SiStat, Production of crops, vegetables, fruits, permanent grassland, Slovenia, annually 2010-2018 – Statistical office Republic of Slovenia (2019).
32. Jäger, H., Peratoner, G., Tappeiner, U. & Tasser, E. Grassland biomass balance in the European Alps: current and future ecosystem service perspectives. *Ecosystem Services* **45**, 101163 (2020).
33. Bayerische Landesanstalt für Landwirtschaft (LfL) Gruber Tabelle zur Fütterung der Milchkühe, Zuchtrinder, Schafe, Ziegen. LfL, Freising-Weihenstephan (2019).
34. Bayerische Landesanstalt für Landwirtschaft (LfL) Gruber Tabelle zur Fütterung in der Rindermast. LfL, Freising-Weihenstephan (2014).
35. Schuster, H. Sojaeinsatz in der Milchviehfütterung. LfL -ITE Sojafeldtag, Wolkertshofen, 12.9.2017; https://www.sojafoerderring.de/ (2017).
36. Landwirtschaftskammer Nordrhein-Westfalen, Tabellen: Futterrationen für Pferde berechnen. Online: https://www.landwirtschaftskammer.de/landwirtschaft/tierproduktion/pferdehaltung/pdf/ (2017).
37. Bayerische Landesanstalt für Landwirtschaft (LfL) Futterberechnung für Schweine. LfL, Freising-Weihenstephan (2020).
38. Bayerische Landesanstalt für Landwirtschaft (LfL) Bayerische Eiweißinitiative: Heimische Eiweißfuttermittel in der Legehennenfütterung. LfL, Freising-Weihenstephan (2017).
39. Bayerische Landesanstalt für Landwirtschaft (LfL) Legehennenfütterung: Einsatz heimischer Futtermittel; Fütterung schnabel-unkupierter Legehennen. LfL, Freising-Weihenstephan (2017).
40. Plesch, P., & Bellof, G. x Rapsextraktionsschrot in der Fütterung von Mastgeflügel. UFOP- Praxisinformation, Berlin (2017).
41. Bayerische Landesanstalt für Landwirtschaft (LfL) Gruber Tabelle zur Fütterung der Milchkühe, Zuchtrinder, Schafe, Ziegen. LfL, Freising-Weihenstephan (2019).
42. Bayerische Landesanstalt für Landwirtschaft (LfL) Gruber Tabelle zur Fütterung in der Rindermast. LfL, Freising-Weihenstephan (2014).
43. Schuster, H. Sojaeinsatz in der Milchviehfütterung. LfL -ITE Sojafeldtag, Wolkertshofen, 12.9.2017; https://www.sojafoerderring.de/ (2017).
44. Landwirtschaftskammer Nordrhein-Westfalen, Tabellen: Futterrationen für Pferde berechnen. Online: https://www.landwirtschaftskammer.de/landwirtschaft/tierproduktion/pferdehaltung/pdf/ (2019).
45. Bayerische Landesanstalt für Landwirtschaft (LfL) Futterberechnung für Schweine. LfL, Freising-Weihenstephan (2020).
46. Bayerische Landesanstalt für Landwirtschaft (LfL) Bayerische Eiweißinitiative: Heimische Eiweißfuttermittel in der Legehennenfütterung. LfL, Freising-Weihenstephan (2018).
47. Bayerische Landesanstalt für Landwirtschaft (LfL) Legehennenfütterung: Einsatz heimischer Futtermittel; Fütterung schnabel-unkupierter Legehennen. LfL, Freising-Weihenstephan (2017)
48. Plesch, P. & Bellof, G. Rapsextraktionsschrot in der Fütterung von Mastgeflügel. UFOP- Praxisinformation, Berlin (2016).
49. Statistik Austria, Agrarstrukturerhebung 2010. Verlag Österreich GmbH, Wien. ISBN 978-3-902791-76-4 (2013).
50. Statistisches Landesamt Baden-Württemberg, Landwirtschaftszählung 2010. Statistisches Landesamt Baden-Württemberg, Stuttgart, 2011 ISSN 1611-2199. / Bayerisches Landesamt für Statistik und Datenverarbeitung (2014) Viehbestände der landwirtschaftlichen Betriebe Bayerns am 1. März 2010. Bayerisches Landesamt für Statistik und Datenverarbeitung, München (2011).
51. Bundesamt für Statistik, Landwirtschaftliche Strukturerhebung. Bundesamt für Statistik, Neuchâtel (2010).
52. Istat, 6° Censimento Generale dell'Agricoltura: Risultati definitivi. Ufficio stampa Istat, Roma (2012).
53. Agreste, Recensement agricole et méthodes de production agricole 2010. Service de la statistique et de la prospective, Paris (2012).
54. Amt für Statistik, Landwirtschaftsstatistik 2010. Amt für Statistik, Vaduz (2010).
55. Statistical Office of the Republic of Slovenia, POPIS kmetijstva 2010 - vsaka kmetija šteje! Statistical Office of the Republic of Slovenia, Ljubljana. ISBN 978-961-239-251-2 (2012).
56. Schneeberger, E. Futtermittel in der Fischproduktion. Garant Tiernahrung, https://www.aqua-garant.at (2017).

57. Reiter, R., Frenzl, B., Schmidt, G., Karl. H. & Manthey-Karl, M. Influence of feed and feeding strategy on fish production and product quality of organic fishery products. *BLE-Forschungsprojekt im Bereich des Bundesprogramms Ökologischer Landbau für den Bereich Aquakultur*, Starnberg (2011).
58. Jarvis, A., Reuter, H.I., Nelson, A. & Guevara, E. Hole-filled SRTM for the globe Version 4, available from the CGIAR-CSI SRTM 90m Database: http://srtm.csi.cgiar.org (2008).
59. EU, Monitoring European climate using surface observations. Copernicus Climate Change Service (C3S). Available at: https://surfobs.climate.copernicus.eu/ 2020 (2022a).
60. EU, Copernicus Climate Change Service: Monitoring European climate using surface observations. https://surfobs.climate.copernicus.eu/ (2022b).
61. Wang, T., Hamann, A., Spittlehouse, D. & Carroll, C. Locally Downscaled and Spatially Customizable Climate Data for Historical and Future Periods for North America. *Plos One* **11**, e0156720 (2016).
62. EU, Corine Land Cover (CLC) 2012, version 18_5. Copernicus Land Monitoring Service (2016).
63. Tappeiner, U., Borsdorf, A. & Tasser, E. (Eds.) Mapping the Alps. Spektrum, Heidelberg (2008).
64. Gobiet, A., Kotlarski, S., Beniston, M., Heinrich, G., Rajczak, J. & x Stoffel, J. 21st century climate change in the European Alps - A review. *Sci. Total Environ.* **493**, 1138-1151 (2014).
